# The Dual Role of Lignin in Fruit Trees: Unraveling Regulatory Networks from Stress Resilience to Quality Control

**DOI:** 10.3390/plants15142244

**Published:** 2026-07-22

**Authors:** Yun Shao, Wenfang Li, Muhammad Mobeen Tahir, Juan Mao, Baihong Chen

**Affiliations:** 1 College of Horticulture, Gansu Agricultural University, Lanzhou 730070, China; liwenf@gsau.edu.cn (W.L.); maojuan@gsau.edu.cn (J.M.); 2 School of Tropical Agriculture and Forestry, Hainan University, Haikou 570228, China; mobeen@hainan.edu.cn

**Keywords:** lignin biosynthesis, fruit trees, chilling injury, stone cell, multi-stress crosstalk, transcriptional regulation, postharvest quality

## Abstract

Lignin deposition in fruit trees represents a fundamental physiological trade-off: essential for structural integrity and stress adaptation, yet excessive or mistimed activation compromises fruit texture, palatability, and market value. This review synthesizes advances in lignin biosynthesis and its multilayered regulation in commercial fruit species. We describe how abiotic (drought, salinity, temperature extremes) and biotic (pathogens, pests) stresses trigger lignification through transcriptional, post-transcriptional, hormonal, and epigenetic mechanisms, centered on the conserved NAC-MYB cascade. This core module integrates WRKY/ERF transcription factors (TFs), microRNA networks, and hormone signaling. Transcriptional programs are further refined by microRNAs, alternative splicing, DNA methylation, histone acetylation, and phytohormone crosstalk (abscisic acid, ABA; jasmonic acid, JA; salicylic acid, SA; and brassinosteroids, BRs). We emphasize molecular crosstalk integrating abiotic and biotic stress signaling via shared TFs, reactive oxygen species (ROS), and epigenetic memory. We critically examine lignification’s dual nature during development and postharvest storage, contributing to desirable traits (stone formation and skin toughness) but also driving defects (stone cell gritty texture and chilling-induced wooliness). Finally, we propose a strategic framework leveraging molecular breeding, targeted gene editing, and precision horticulture to fine-tune lignification, enabling climate-resilient cultivars without compromising fruit quality.

## 1. Introduction

Fruit trees are long-lived perennial woody plants that underpin global horticulture by providing essential nutrition, economic value, and ecological stability [1]. Throughout their extended lifespans, they encounter a wide spectrum of environmental challenges—including drought, salinity, temperature extremes, heavy metal toxicity, and diverse biotic stresses from pathogens and pests [1,2,3]. With climate change intensifying the frequency and severity of these stresses, understanding the molecular mechanisms underlying fruit tree adaptation has become critical for sustaining agricultural productivity and ensuring long-term resilience.

Fruit trees adopt a variety of defense mechanisms; however, lignification—the deposition of lignin into secondary cell walls—stands out as a key defense mechanism [2,4]. Lignin is a complex phenolic polymer derived from the monolignols p-coumaryl (H), coniferyl (G), and sinapyl (S) alcohol, synthesized via the phenylpropanoid pathway [4,5,6]. Beyond providing mechanical strength, lignin plays important roles in water transport, wound sealing, and resistance to both abiotic and biotic stress conditions [4,6,7,8]. However, this protective function comes with a cost: when dysregulated, lignification can negatively impact fruit quality, leading to undesirable textural attributes, such as stone cell gritty texture, mealiness, or excessive firmness [9,10,11].

Transcriptional, post-transcriptional, hormonal, and epigenetic mechanisms constitute complex regulatory systems that coordinate lignin biosynthesis in major fruit trees, apple (*Malus domestica*), pear (*Pyrus* spp., primarily *P. bretschneideri* and *P. pyrifolia*), citrus (*Citrus* spp., primarily *C. maxima* and *C. sinensis*), peach (*Prunus persica*), loquat (*Eriobotrya japonica*), and grape (*Vitis vinifera*) [12,13,14,15,16]. Stress-induced lignification, often enriched in guaiacyl (G) units, enhances tissue rigidity and contributes significantly to protection against diverse environmental challenges [2,17]. This review synthesizes current advances in understanding lignin biosynthesis, its complex regulatory networks, and its dual functional roles in stress adaptation and fruit quality management. By integrating findings across multiple fruit tree species, we aim to provide a comprehensive framework that bridges fundamental molecular insights with practical applications, ultimately supporting future research and guiding breeding strategies for developing resilient, high-quality cultivars.

This review is based on a comprehensive literature search of PubMed, Web of Science, and Google Scholar for peer-reviewed articles published between 2002 and 2026. Search terms included combinations of ‘lignin biosynthesis,’ ‘fruit trees,’ ‘stress tolerance,’ and ‘postharvest quality,’ with reference lists of key papers further examined for additional studies.

## 2. Lignin Biosynthesis and Regulation in Fruit Trees

Lignin biosynthesis proceeds through the phenylpropanoid pathway, producing the monolignols H, G, and S alcohols, which polymerize to form lignin [4,6,18]. These monolignols form the structural backbone of secondary cell walls and contribute to mechanical strength, water transport, and defense [4,6,19,20]. Lignin content and composition in fruit trees are tightly regulated by complex biosynthetic, transcriptional, post-transcriptional, hormonal, and epigenetic networks, which collectively ensure proper developmental progression, stress adaptation, and fruit quality formation.

### 2.1. The Lignin Biosynthetic Pathway

The lignin biosynthetic pathway involves core enzymes, including Phenylalanine Ammonia-Lyase (PAL), Cinnamate 4-Hydroxylase (C4H), 4-Coumarate:CoA Ligase (4CL), Cinnamoyl-CoA Reductase (CCR), Cinnamyl Alcohol Dehydrogenase (CAD), Caffeic Acid O-Methyltransferase (COMT), and Ferulate 5-Hydroxylase (F5H) [4,6]. Branch-point enzymes such as *p*-Coumaroyl Shikimate/Quinate Hydroxycinnamoyltransferase (HCT), Caffeoyl-CoA O-Methyltransferase (CCoAOMT), and *p*-Coumarate 3-Hydroxylase (C3H) direct metabolic flux toward specific monolignols, while endoplasmic reticulum (ER)-anchored cytochrome P450s (C4H, C3H, and F5H) enhance pathway efficiency. The activity of these enzymes can be modulated under stress [21,22,23,24].

Following cytoplasmic synthesis, monolignols must be exported to the apoplast, a step primarily mediated by ATP-binding cassette (ABC) transporters, particularly the ABCG subfamily [25]. Although *AtABCG29* remains the only genetically characterized monolignol transporter in Arabidopsis (*Arabidopsis thaliana*) [26], accumulating evidence supports its functional conservation in fruit trees. In pomegranate (*Punica granatum*), *PgABCG9* negatively regulates lignin accumulation by sequestering monolignols, with its expression inversely correlated with seed hardness [27]; in apple, *MdABCG25* is stress-inducible [28], and transcriptomic data reveal multiple ABC transporters upregulated under postharvest stress [29]; in pear, ABCG family members correlate with lignin biosynthesis during russet formation [30].

Concurrent with transport, a monolignol glucosylation–deglucosylation cycle precisely regulates the timing of lignin deposition. Monolignols are reversibly glucosylated by UDP-glycosyltransferases (UGTs) into stable glucosides (e.g., coniferin), which prevents premature polymerization in the cytosol; these glucosides are later hydrolyzed by apoplastic β-glucosidases (BGLUs) to release active monolignols at the cell wall [31]. This cycle is functionally relevant in fruit trees: in pear, systematic analysis identified *PbBGLU16* as a key player in stone cell lignification, with its overexpression increasing both lignin content and stone cell number [32]. Likewise, in poplar (*Populus* spp), *UGT72* family members glycosylate monolignols, and *ugt72b37* mutants show altered lignin content, indicating evolutionary conservation of this regulatory mechanism in woody species [33,34].

Finally, the oxidative coupling of monolignols into lignin polymers is catalyzed by peroxidases (PRXs) and laccases (LACs), whose coordinated activity constitutes a major terminal control point for lignin deposition [35]. PRXs utilize hydrogen peroxide (H_2_O_2_), whereas LACs use molecular oxygen, directly linking lignification to the cellular redox environment [36]. H_2_O_2_ is primarily generated by plasma membrane NADPH oxidases (RBOHs), connecting reactive oxygen species (ROS) production with the onset of polymerization [37]. Collectively, this multi-layered biosynthetic sequence—from ER-anchored catalysis and ABC-mediated transport, through glucoside storage and release, to redox-dependent oxidative polymerization—orchestrates lignin deposition with high spatiotemporal fidelity, directly influencing key fruit-quality attributes, such as stone cell formation, seed hardness, and stress-induced russeting. Supplementary Table S1 provides a complete list of abbreviations, and Supplementary Table S2 compiles the reported lignin-related genes across the fruit tree species discussed in this review.

### 2.2. Transcriptional Regulation

Across fruit trees, *CAD*, *CCR*, and *PAL* frequently display strong stress-responsive expression patterns, with *CAD* particularly critical for drought and salt tolerance by sustaining monolignol supply for vascular reinforcement. Genome-wide analyses in apple reveal multiple lignin-related genes co-induced under diverse stress conditions [13,38,39,40]. The conserved NAC–MYB cascade forms the core framework: upstream NAC transcription factors (TFs) activate downstream MYB regulators, which orchestrate transcription of lignin biosynthetic genes [12,38,41].

In apple, NAC TFs, such as *MdSND1*, initiate lignin biosynthesis and enhance salt and osmotic stress tolerance by activating secondary wall–associated MYBs, including *MdMYB46* and *MdMYB83* [13,40]. Overexpression of *MdMYB46* strengthens secondary wall formation by directly activating multiple lignin biosynthetic genes [42]. Similar regulatory logic is observed in other fruit trees: *CgNAC043* in pomelo activates *CgMYB46* and directly targets *CgCCoAOMT* and *CgC3H* to drive juice sac lignification [43], whereas *PbrNSC* in pear promotes stone cell lignification by regulating *PbrMYB169*, *Pbr4CL4*, and *PbrLAC4*, demonstrating the regulatory diversity of this conserved pathway [44]. The pear regulatory network has been further expanded: *PbrMYB24* acts as an additional activator of lignin and cellulose biosynthesis [45], whereas a *PbAGL7*-*PbNAC47*-*PbMYB73* transcriptional complex coordinately activates *PbC3H1* and *PbHCT17* to drive lignification [46].

While NAC–MYB cascades predominantly act as positive regulators, lignification is also modulated by transcriptional repressors that prevent unnecessary metabolic expenditure or structural defects. R2R3-MYB repressors—orthologs of Arabidopsis *MYB4*, *MYB7*, and *MYB32*—compete with activator MYBs for AC-rich cis-elements, thereby suppressing phenylpropanoid flux under normal growth conditions [47,48]. The activity of such repressors is crucial for maintaining metabolic and structural homeostasis, preventing inappropriate lignification until developmental or stress cues activate the biosynthetic pathway. This dynamic balance between activators and repressors ensures precise spatiotemporal control of lignin biosynthesis.

Other TF families, including WRKY and ERF, fine-tune lignification. WRKY TFs contribute to developmental and stress-induced lignin deposition. *PbWRKY24* promotes russet skin lignification in pear [49], *PlWRKY29* regulates lignin in herbaceous peony (*Paeonia lactiflora*) under stress [50], and VvWRKY2 binds the VvC4H promoter to mediate stress-responsive lignification in grapevine (*Vitis vinifera*) [51]. ERFs also participate in stress-responsive lignification. For example, in apple, *MdERF114* binds the GCC-box of the *MdPRX63* promoter, enhancing PRXs-mediated lignin polymerization and increasing resistance to *Fusarium solani* [52]. Together, NAC, MYB, WRKY, and ERF TFs—integrating activators and repressors—constitute a robust framework that aligns lignin biosynthesis with development, fruit quality, and stress responses.

### 2.3. Hormonal Regulation

Phytohormones are key integrators of developmental and environmental signals that collectively shape lignification patterns in fruit trees. Hormone perception is generally initiated by specialized receptor complexes—pyrabactin resistance/pyr-like/regulatory component of ABA receptor (PYR/PYL/RCAR) for abscisic acid (ABA), transport inhibitor response 1/auxin signaling f-box (TIR1/AFB) for auxin, coronatine insensitive 1 (COI1) for jasmonic acid (JA), and brassinosteroid insensitive 1 (BRI1) for brassinosteroids (BRs)—which convert extracellular cues into transcriptional responses [53,54,55]. These signaling systems regulate lignin biosynthesis by modulating master TFs and pathway enzymes, allowing fruit trees to dynamically adjust cell wall structure according to developmental needs and stress severity.

BRs coordinate cell expansion and secondary cell wall formation through direct interactions between BR-responsive TFs and lignification regulators. The BR-activated TFs BRI1-EMS-SUPPRESSOR 1 (BES1) and BRASSINAZOLE-RESISTANT 1 (BZR1) interact with MYB46 and NAC master switches, modulating their activity to balance rapid growth with controlled wall thickening [56,57]. In fruit trees, this coordination is likely crucial during early fruit enlargement, when excessive lignification could hinder growth. Evidence from non-fruit species indicates that BRs can suppress premature secondary wall formation, suggesting a conserved mechanism that may also operate in fruit crops. Conversely, gibberellins (GAs) generally act as negative regulators of lignification. In pear, GA signaling reduces lignin-rich stone cell formation via DELLA-mediated repression of lignification-associated TFs, and exogenous GA treatment is commonly used in horticulture to improve fruit texture by lowering stone cell content [14,58].

Under abiotic stress, ABA emerges as a central inducer of lignification. ABA signaling, initiated through PYR/PYL/RCAR receptors and transmitted via PROTEIN PHOSPHATASE 2C (PP2C) inhibition and SNF1-RELATED PROTEIN KINASE 2 (SnRK2) activation, upregulates NAC and MYB TFs as well as core biosynthetic genes, such as *PAL* and *CAD* [2,40,59]. ABA-induced lignification reinforces vascular tissues, enhances apoplastic barrier formation, and improves mechanical stability under drought and salinity—key adaptations for long-lived perennial fruit trees facing repeated stress episodes.

In biotic stress responses, JA and salicylic acid (SA) constitute the principal hormonal drivers of defense lignification. JA perception through the COI1–JAZ co-receptor complex leads to JASMONATE ZIM-DOMAIN (JAZ) degradation and activation of MYB and WRKY TFs that stimulate lignin deposition at wound sites or during herbivory [54,60]. The activation of MYB TFs by jasmonate signaling is well-established; for instance, in *Panax ginseng*, the MeJA-responsive *PgMYB2* positively regulates secondary metabolite biosynthesis [61], illustrating a conserved regulatory module that likely operates in fruit tree lignification pathways. SA contributes to pathogen-triggered lignification by enhancing phenylpropanoid gene activation and strengthening cell wall–based defenses and interacts synergistically or antagonistically with JA depending on pathogen lifestyle (necrotrophic vs. biotrophic), enabling tailored defense responses [20,62].

Hormonal crosstalk operates at multiple regulatory levels to integrate growth, defense, and metabolic priorities. Shared TFs—particularly NACs and MYBs—serve as convergence nodes where developmental hormones (auxin and BRs) and stress hormones (ABA and JA) collectively determine lignification output [63]. Co-regulation of biosynthetic genes enables multiple hormones to influence the same enzymatic steps, while metabolic competition within the phenylpropanoid pathway imposes trade-offs: carbon diverted toward stress-induced lignin synthesis may reduce flux toward flavonoids or tannins [64]. This multilayered hormonal integration allows fruit trees to fine-tune lignification in fluctuating environments, ensuring appropriate mechanical reinforcement without compromising growth or fruit quality.

### 2.4. Post-Transcriptional and Epigenetic Regulation

Lignification is further refined by post-transcriptional and epigenetic mechanisms that complement the transcriptional networks. These layers of regulation allow fruit trees to precisely adjust lignin deposition in response to developmental cues and rapidly changing environmental conditions. MicroRNAs (miRNAs) are key post-transcriptional regulators that influence both TFs and lignin pathway genes. Among them, miR397 is a conserved regulator that targets *LAC* genes involved in monolignol polymerization, modulating lignin content and composition in pear and potentially other fruit trees [62,65]. Evidence from model plants suggests other miRNAs may also be involved; for instance, in Arabidopsis, miR858 targets R2R3-MYB TFs that activate lignin biosynthetic genes, and miR164 modulates NAC-domain master regulators [66,67]. Their potential roles in fruit trees warrant future investigation.

Alternative splicing provides another versatile layer of post-transcriptional regulation by generating protein isoforms with altered enzymatic activities, subcellular localizations, or stress sensitivities. In the woody model poplar, *PAL* genes undergo stress-responsive alternative splicing, producing truncated proteins that may act as dominant-negative competitors [7,68]. Likewise, *4CL* genes in potato (*Solanum tuberosum*) exhibit extensive alternative splicing, producing multiple functional variants [69,70]. While large-scale studies in fruit trees are needed, these findings in other species highlight a likely conserved mechanism for the rapid reprogramming of metabolic pathways, including lignin biosynthesis under stress. Despite these findings in model woody species, direct evidence for stress-responsive alternative splicing of lignin genes in fruit trees remains scarce. This represents a significant knowledge gap, as the perennial nature and long reproductive cycles of fruit trees may favor splicing-mediated regulatory flexibility as an adaptive strategy.

Protein stability regulation through the ubiquitin-proteasome system (UPS) enables rapid adjustment of lignin biosynthetic enzyme abundance without requiring transcriptional changes. E3 ubiquitin ligases recognize degradation motifs (degrons) and target proteins for proteasomal breakdown [71]. In apple, for example, the TF *MdMYB88* is regulated through UPS-mediated degradation to fine-tune phenylpropanoid metabolism, including lignin biosynthesis [72]. In Arabidopsis, core enzymes, such as PAL, are post-translationally stabilized by environmental cues, such as light, which protects PAL from degradation and promotes phenylpropanoid flux [73]. Additional post-translational modifications—including phosphorylation—can expose or mask degrons, modulating protein stability and integrating external signals with metabolic turnover [7].

Epigenetic modifications also play a crucial role in shaping lignification patterns. In fruit trees, although mechanistic studies directly linking specific epigenetic marks to lignin deposition are still emerging, evidence confirms that DNA methylation is a powerful regulator of developmental traits involving secondary cell wall metabolism. For instance, a study on pear domestication revealed that selected global increases in DNA methylation are associated with the downregulation of phenylpropanoid biosynthesis genes and contribute to fruit ripening timing [74]. This demonstrates that DNA methylation can repress metabolic pathways in a development-specific manner. Such epigenetic mechanisms are likely involved in the spatial control of lignin deposition—such as distinguishing lignified stone cells from parenchyma—though this specific spatial link awaits further validation.

In loquat, direct evidence links DNA methylation to chilling-induced lignification: the *EjNAC5* promoter contains a differentially methylated region, and its methylation level is negatively correlated with *EjNAC5* transcript abundance; in red-fleshed ‘Dahongpao’ fruit, which are prone to chilling-induced lignification at 0 °C, *EjNAC5* promoter methylation is lower than in white-fleshed ‘Baisha’ fruit, which do not undergo chilling lignification [75].

Histone modifications provide an additional dynamic layer of control, where acetylation generally promotes gene expression by loosening chromatin, and deacetylation condenses it to repress transcription. In poplar, histone deacetylases, such as *PtrHDA907* and *PtrHDA908*, interact with TFs to reduce acetylation at lignin-related gene promoters, thereby modulating lignification during development and under stress [16,76]; their potential roles in fruit trees warrant future investigation. Collectively, these findings suggest that perennial woody species, including fruit trees, rely on epigenetic plasticity to fine-tune complex biosynthetic processes like lignification across developmental stages and environmental fluctuations without permanent genetic changes. Recent studies have provided direct fruit-tree evidence for this regulation. In pear, a landmark domestication study revealed that global increases in DNA methylation are significantly correlated with the downregulation of phenylpropanoid biosynthesis genes, contributing to early fruit ripening [74].

## 3. Lignin and Abiotic Stress Tolerance in Fruit Trees

**Figure 1:**
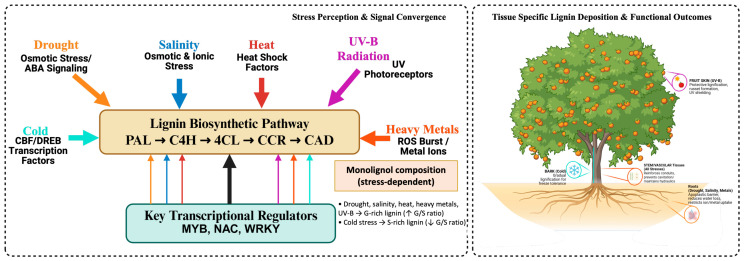
Lignin-mediated responses to abiotic stresses in fruit trees. Major abiotic stresses, including drought, salinity, temperature extremes, heavy metal toxicity, and UV-B radiation, are perceived through distinct primary signaling pathways involving abscisic acid (ABA), reactive oxygen species (ROS), UV RESISTANCE LOCUS 8 (UVR8), heat shock factors (HSFs), and C-repeat binding factors (CBFs). These stress signals converge on a shared set of key transcription factors (TFs), particularly members of the MYB, NAC, and WRKY families, which coordinately regulate the core phenylpropanoid and monolignol biosynthesis pathway, including *PAL*, *C4H*, *4CL*, *CCR* and *CAD*. Stress-associated changes in lignin deposition, often accompanied by stress-dependent shifts in monolignol composition (G/S ratio, annotated in the bottom-right panel), promote targeted structural reinforcement in specific tissues: enhanced lignification of root apoplastic barriers limits water and ion loss; reinforcement of xylem vessels improves resistance to cavitation; and protective lignification of fruit skin mitigates UV-induced damage. Collectively, these lignification-mediated adaptations enhance whole-plant resilience under adverse environmental conditions. This figure summarizes the abiotic stress responses discussed in Section 3, providing a visual framework for understanding how diverse environmental challenges trigger lignin biosynthesis through shared regulatory networks.

While the above regulatory mechanisms operate under normal developmental conditions, fruit trees must dynamically deploy these programs under environmental stress. Fruit trees face a wide range of abiotic stresses throughout their lifespan, including drought, salinity, extreme temperatures, heavy metal toxicity, and ultraviolet (UV) radiation. These stresses trigger complex physiological and molecular responses, with lignification serving as a central adaptive mechanism that reinforces structural integrity, modulates water transport, and mitigates cellular damage [2,19]. Under stress conditions, fruit trees often show increased lignin deposition in roots, stems, and vascular tissues, accompanied by upregulation of lignin biosynthetic genes and shifts in monolignol composition [17]. Figure 1 illustrates how abiotic stress signaling converges on lignin biosynthesis to enhance stress tolerance in fruit trees. Table 1, in turn, summarizes stress-specific responses across different fruits, including key genes/regulators, tissue-specific responses, major findings, and corresponding references.

### 3.1. Drought Stress and Lignin Accumulation

Drought is among the most critical constraints on fruit tree productivity. Water deficit induces extensive lignification in roots, stems, and vascular tissues, enhancing hydraulic conductivity and maintaining structural stability under low turgor pressure [2,77]. At the molecular level, drought upregulates key lignin biosynthetic genes, such as *PAL*, *C4H*, *4CL*, *CCR*, and *CAD*, resulting in increased lignin content and altered G/S monolignol ratios [77,78]. This transcriptional response is coordinated by a complex signaling network. For instance, in melon (*Cucumis melo*), drought-induced accumulation of ABA, H_2_O_2_, and JA converges to differentially upregulate specific isoforms of *CmCADs* and other pathway genes (*PAL*, *POD*, and *LAC*), ensuring robust lignin biosynthesis for stem reinforcement [79]. In apple, enhanced expression of *MdPAL*, *MdC4H*, and *MdCAD* promotes lignin deposition in xylem vessels, improving drought tolerance [13].

Beyond structural support, drought-induced lignification strengthens apoplastic barriers in the root endodermis and exodermis, reducing radial water loss and preventing backflow under low soil water potential [80,81]. Lignified xylem conduits resist cavitation and embolism, maintaining efficient water transport [8,82]. MYB and NAC TFs coordinate this response; for example, *MdMYB46* activates lignin biosynthetic genes under osmotic stress, enhancing cell wall rigidity and minimizing water loss in transgenic apple plants [43]. Systematic reviews of the transcriptional framework governing drought-induced lignification have underscored the central role of NAC-MYB modules in integrating stress signals with lignin biosynthetic flux [83].

This regulatory system is fine-tuned by epigenetic and post-transcriptional mechanisms. Epigenetic modifications, such as histone acetylation mediated by histone deacetylases, facilitate rapid transcriptional activation of lignin genes during drought [76]. Recently, a key post-transcriptional control layer has been identified: N6-methyladenosine (m^6^A) RNA modification. In apple, the m^6^A writer, *MdMTA*, stabilizes and enhances the translation of mRNAs for lignin biosynthesis and ROS scavenging under drought, directly linking this epitranscriptomic regulation to lignification and stress tolerance [84].

Importantly, ABA-mediated lignification is not limited to drought adaptation but also influences fruit quality under physiological stress. For instance, in pear, exogenous ABA induces corking disorder by upregulating lignin biosynthesis through the *PbrMYB8*–*PbrMYB169* transcriptional module, which co-activates lignin pathway genes [85]. This stress-responsive module differs from the developmental stone-cell pathway described later (Section 6.1), where *PbrMYB169* functions independently to activate lignin genes during early fruit development; the ABA-inducible recruitment of *PbrMYB8* as a partner thus represents a stress-specific regulatory configuration that drives pathological lignification in fruit tissues. This highlights how ABA–MYB regulatory networks can drive lignin accumulation across different tissues and stress contexts, from drought-hardening in woody vasculature to post-harvest disorders in fruit.

### 3.2. Salinity Stress and Structural Reinforcement

Salinity imposes osmotic and ionic stress, disrupting cellular homeostasis and weakening tissue integrity. Lignin biosynthesis is strongly induced under salt stress, reinforcing cell walls, restricting ion influx, and maintaining vascular organization [86,87]. In citrus, salt stress induces expression of *CsPAL*, *CsC4H*, and *Cs4CL*, enhancing lignin accumulation in roots and stems, which is proposed as a stress tolerance mechanism [88]. The reinforcement of cell walls through lignification is a recognized adaptive response to salt stress [87].

Functionally, lignification in root apoplastic barriers limits Na^+^ uptake, protecting aerial tissues from ionic toxicity, as demonstrated in crops such as rice (*Oryza sativa*) [89]. The molecular initiation of this response involves stress perception at the cell surface. In sweet cherry (*Prunus avium*), the lectin receptor-like kinase *PaLectinL7*, a plasma membrane-localized protein, is upregulated by salt stress. Its overexpression enhances salt tolerance and leads to increased lignin content and activity of key enzymes like CAD and COMT, positioning it as an upstream regulator of the lignification program [90].

Transcriptional regulation involves stress-specific factors; in apple, *MdSND1* activates downstream lignin genes via the NAC–MYB cascade, cooperating with *MdMYB46* and *MdMYB83* to enhance lignin deposition [13,40]. This transcriptional control can be coupled with direct post-translational modulation of the lignin machinery. PaLectinL7 physically interacts with and phosphorylates PaCAD1, a key lignin biosynthetic enzyme, suggesting a dual-layer regulation where membrane-mediated signaling can rapidly fine-tune lignin synthesis through enzyme activity alongside gene expression [90]. Salinity also modulates lignin composition, often increasing G-lignin content and the G/S ratio through regulation of enzymes such as F5H and COMT, which enhances cell wall rigidity and hydrophobicity, improving salt stress tolerance [91].

### 3.3. Temperature Extremes and Lignin Dynamics

Both heat and cold stress trigger lignification, though with differing dynamics and functional outcomes. Heat stress rapidly increases *PAL* and *CAD* activity, reinforcing cell walls to prevent thermal expansion damage and cellular dehydration [2,19]. In pear, elevated temperatures upregulate *PAL* and *CCR*, increasing lignin in the endocarp [9]. Cold stress, in contrast, promotes gradual lignification in overwintering organs, such as bark and dormant buds. In apple, cold acclimation is associated with the sustained expression of phenylpropanoid genes, such as *MdC4H* and *MdCAD*, which contributes to a gradual increase in lignin deposition in the bark. This lignification enhances mechanical strength and freeze tolerance, likely by mitigating ice crystal-induced cell wall damage [13].

Molecular regulation integrates thermal sensing with secondary wall biosynthesis. Heat shock factors (HSFs) interact with MYB and NAC TFs to coordinate lignin synthesis under high temperatures [92]. In grapevine, the heat-induced expression of *VvHSFA2* [93] suggests this regulatory module may be conserved. Conversely, cold-induced lignification is coordinated by CBF/DREB TFs that interact with MYBs to regulate lignin biosynthetic genes [94]. Beyond these transcriptional cascades, membrane-initiated signaling plays a key role. In apple, the FERONIA family receptor-like kinase *MdMRLK2* is rapidly induced by cold. *MdMRLK2* overexpression enhances cold tolerance by promoting the accumulation of lignin, cellulose, and hemicellulose, likely through the suppression of cell wall-degrading enzymes. Furthermore, *MdMRLK2* interacts with the *MdMYBPA1* to regulate anthocyanin biosynthesis, illustrating a coordinated regulation of multiple cold-adaptive pathways [95]. Temperature stress also affects monolignol composition: heat favors G-lignin via increased *C3H* and *HCT* activity, while cold promotes S-lignin through *F5H* and *COMT* upregulation [2].

### 3.4. Heavy Metal Toxicity and Oxidative Stress

Heavy metals, including cadmium, lead, copper, and aluminum, disrupt metabolism and generate oxidative stress. Lignification mitigates toxicity by immobilizing metals and limiting translocation to edible organs [96,97]. In apple, aluminum stress induces *MdPAL*, *Md4CL*, and *MdCCR*, promoting lignin deposition in root apoplasts that bind Al^3+^ and restrict symplastic entry [13].

ROS signaling closely links heavy metal stress to lignification. Metal-induced ROS activate lignin biosynthetic genes, while PRXs and LACs catalyze monolignol polymerization for rapid deposition [96,98,99]. Phenolic intermediates also act as antioxidants, scavenging ROS and mitigating oxidative damage [100].

### 3.5. UV-B Radiation and Protective Lignification

UV-B radiation (280–315 nm) induces oxidative stress and triggers protective responses, including enhanced phenylpropanoid metabolism and lignin deposition, particularly in fruit skin. This response is mediated by the UV RESISTANCE LOCUS 8 (UVR8) photoreceptor, which activates TFs controlling phenylpropanoid genes [101]. In apple, UV-B regulates fruit development and flavonoid (anthocyanin/flavonol) accumulation through a transcriptional cascade involving *HY5*, *MYB10*, and *MYB22*, with the flavonol synthase (FLS) promoter being a key integration point [102]. Crucially, the same signal can also drive lignification. Studies on apples show that while moderate UV-B irradiation (≥1 W m^−2^ s^−1^) effectively promotes anthocyanin synthesis, higher intensities (5 W m^−2^ s^−1^) can lead to lignin hyperaccumulation, suggesting a temporal hierarchy in pathway activation where anthocyanin genes respond more rapidly than lignin biosynthetic genes [103].

This lignification response contributes directly to skin properties. In pear, UV-B-induced lignification is mediated by the *PbWRKY24-PbPRX4* regulatory module and contributes to russet formation, with cultivar-dependent differences in sensitivity [49]. Importantly, russeting and lignification in fruit peel are regulated by a broader spectrum of light. In ‘Cuiguan’ pear, fruit bagging with materials that transmit higher proportions of red/far-red (600–800 nm) and UV-A (350–400 nm) light correlates with increased expression of lignin-biosynthesis genes (*PAL*, *C4H*, *4CL*, *CAD*, and *CCR*), higher lignin content, and more severe russeting. Bags that filter these wavelengths reduce russeting, suggesting a role of light quality in regulating peel lignification [104]. Therefore, light quality is a decisive environmental regulator directing phenylpropanoid flux toward lignin, fundamentally shaping pericarp development and skin texture.

### 3.6. Comparative Synthesis: Shared Versus Stress-Specific Lignification Responses

Several common themes emerge across diverse abiotic stresses. ROS and hormonal signals (ABA, JA, and SA) act as core integrators of stress perception, with their signaling cascades intersecting with phenylpropanoid metabolism and contributing to downstream adaptive responses [105].

Stress-specific features are equally important. For instance, salinity promotes root lignification that restricts apoplastic Na^+^ influx [89]; heavy metals induce lignification that helps immobilize toxic ions in the apoplast [96]; temperature extremes broadly activate phenylpropanoid pathways [94]; and UV-B specifically upregulates flavonoid biosynthesis in fruit skin, a key branch of phenylpropanoid metabolism [102]. Meanwhile, developmental lignification establishes foundational apoplastic diffusion barriers in roots [80], while drought stress is broadly associated with enhanced root and xylem lignification.

A unified dose-response hypothesis can be proposed: low to moderate stress activates protective lignification, enhancing tolerance without major quality penalties; moderate to high stress triggers excessive lignification, compromising tissue integrity (e.g., fruit texture); and extreme stress leads to dysregulated, ectopic lignification, causing structural disorders. The threshold between protective and detrimental lignification is likely governed by stress duration, tissue-specific competence, and cultivar genetic background. Quantifying these thresholds across species and cultivars represents a critical priority for precision stress management.

**Table 1:** Lignin-mediated responses to abiotic stresses in fruit trees.

Stress Type	Fruit Name	Key Genes/Regulators	Tissue Response	Key Findings	References
Drought	Apple	*MdSND1*, *MdMYB46*, *MYB83*	Whole plant	*MdSND1* activates the NAC-MYB cascade, enhancing lignin deposition and improving osmotic stress tolerance	[40]
	Apple	*MdMTA* (m^6^A writer)	Whole plant	*MdMTA* stabilizes lignin and ROS-scavenging transcripts via m^6^A modification, promoting lignin deposition and drought tolerance	[84]
	Melon	*CmCAD1*, *CmCAD2*, *CmCAD3*, *CmCAD5*, *CmPAL*, *CmPOD*, *CmLAC*	Stems	ABA, H_2_O_2_, and JA signaling converge to upregulate CAD isoforms and lignin genes, coordinating stem lignification under drought	[79]
	Pear	*PbrMYB8-PbrMYB169* module	Fruit cork tissue	ABA induces the MYB8-MYB169 heterodimer, which co-activates lignin genes and drives pathological corking disorder lignification	[85]
Salinity	Citrus	*CsPAL*, *CsC4H*, *Cs4CL*	Roots, stems	Salt stress induces phenylpropanoid gene expression, leading to enhanced lignification as a proposed tolerance mechanism	[88]
	Apple	*MdSND1*, *MdMYB46*, *MdMYB83*	Whole plant	Activation of the NAC-MYB cascade promotes lignin deposition, thereby enhancing salt tolerance	[40]
	Sweet cherry	*PaLectinL7*, *PaCAD1*	Roots	PaLectinL7 promotes lignin deposition and phosphorylates PaCAD1, coordinating transcriptional and post-translational regulation for salt tolerance	[90]
Temperature extremes	Pear	Phenylpropanoid pathway (implied)	Fruit endocarp	Heat activates the phenylpropanoid pathway, increasing endocarp lignin and contributing to stone cell development	[9]
	Apple	*MdMRLK2*, *MdMYBPA1*	Whole plant	Cold-induced *MdMRLK2* promotes lignin and cell wall accumulation and interacts with MdMYBPA1, coordinating cold tolerance and secondary metabolism	[95]
UV-B radiation	Apple	Lignin: *PAL*, *4CL*, *CCR*, *CAD*; anthocyanin: *UFGT*, *DFR*	Fruit peel	Moderate UV-B promotes anthocyanin, whereas high-intensity UV-B induces lignin hyperaccumulation, indicating a dose-dependent flux shift	[103]
	Pear	*PbWRKY24-PbPRX4* module	Fruit peel	*PbWRKY24* upregulates *PbPRX4*, driving lignin accumulation and contributing to russet formation and altered texture	[49]
	Pear	*PAL*, *C4H*, *4CL*, *CAD*, *CCR*	Fruit peel	Light quality (red/far-red and UV-A) regulates lignin gene expression; specific filtering reduces lignin accumulation and inhibits russeting	[104]

Abbreviations: ABA, abscisic acid; CAD, cinnamyl alcohol dehydrogenase; CCR, cinnamoyl-CoA reductase; C4H, cinnamate 4-hydroxylase; 4CL, 4-coumarate:CoA ligase; DFR, dihydroflavonol 4-reductase; H_2_O_2_, hydrogen peroxide; JA, jasmonic acid; LAC, laccase; m^6^A, N6-methyladenosine; PAL, phenylalanine ammonia-lyase; POD, peroxidase; PRX, class III peroxidase; ROS, reactive oxygen species; UFGT, UDP-glucose:flavonoid 3-O-glucosyltransferase; UV-A, ultraviolet A; UV-B, ultraviolet B.

## 4. Lignin and Biotic Stress Tolerance in Fruit Trees

**Figure 2:**
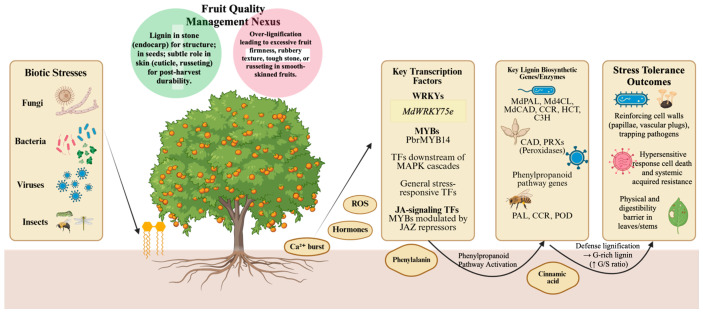
Biotic stress–induced lignin biosynthesis and its implications for stress tolerance and fruit quality in fruit trees. Biotic stresses, including fungi, bacteria, viruses, and insect herbivores, activate early defense signaling events, such as reactive oxygen species (ROS) production, phytohormone signaling, and Ca^2+^ influxes, which converge on mitogen-activated protein kinase (MAPK) cascades. These pathways stimulate downstream transcription factors (TFs), primarily WRKY and MYB families, to induce the phenylpropanoid and lignin biosynthetic pathway. Phenylalanine is converted via cinnamic acid through coordinated regulation of core enzymes (PAL, C4H, 4CL, HCT, C3H, CCR, and CAD) and polymerization enzymes (laccases and peroxidases), leading to spatially regulated lignin deposition. Enhanced lignification reinforces cell walls, restricts pathogen spread, and contributes to local and systemic defense responses. While regulated lignification supports fruit structural integrity and postharvest durability, excessive lignin accumulation can negatively affect fruit texture and sensory quality. Defense lignification is characterized by G-rich lignin (↑ G/S ratio), as annotated in the figure, which enhances wall rigidity and resistance to pathogen enzymatic degradation.

Beyond physical stresses, the same lignification machinery forms essential barriers against biological threats from pathogens and herbivores. Building on the regulatory mechanisms described earlier, lignification is a crucial defense strategy against diverse biotic threats. Pathogens and herbivorous pests pose major challenges to fruit tree health and productivity. Lignin contributes to both preformed and induced defenses, forming physical barriers against pathogen penetration while integrating with active defense signaling networks [20]. Upon pathogen attack or insect herbivory, fruit trees rapidly deposit lignin at infection or damage sites—a process termed “defense lignification,” which restricts pathogen spread and reinforces cell walls [20,106]. This section details the molecular and physiological mechanisms underlying lignin-mediated resistance to fungal, bacterial, viral, and insect challenges. Figure 2 illustrates biotic stress–induced lignin biosynthesis and its implications for stress tolerance and fruit quality in fruit trees. Table 2, in turn, summarizes stress type–specific regulatory mechanisms across different fruit crops, including key genes and regulators, tissue-level responses, principal findings, and supporting references.

### 4.1. Fungal Pathogen Resistance and Defense Lignification

Fungal pathogens, including fire blight, apple scab, pear rust, and brown rot, trigger rapid lignin deposition at infection sites, forming physical barriers that limit hyphal penetration and pathogen colonization [20,107]. In apple, defense against *Alternaria alternata* involves a well-defined transcriptional cascade. The JA-induced TF *MdWRKY75e* is a central regulator; it directly binds to the promoter of the laccase gene *MdLAC7*, upregulating its expression to drive lignin biosynthesis and cell wall thickening, thereby enhancing mechanical resistance and lesion confinement [108]. This mechanism complements the localized upregulation of other core lignin biosynthetic genes (e.g., *MdPAL*, *Md4CL*, and *MdCAD*) observed during infection [109]. Grapevine resistance to *Botrytis cinerea* has been associated with phenylpropanoid pathway activation and *CCR* upregulation, strengthening cell walls against fungal invasion, as reviewed by Rahman, et al. [110]. Similarly, in postharvest litchi (*Litchi chinensis*) fruit, the resistant variety ‘Heiye’ exhibits increased lignin accumulation in the pericarp upon infection with *Peronophythora litchii*, mediated by upregulation of *C4H*, *COMT*, *CAD*, and *POD* genes. Enhanced ROS scavenging via SOD and CAT further contributes to defense, highlighting the coordinated roles of lignin biosynthesis and antioxidant metabolism in restricting pathogen progression compared to the susceptible variety ‘Guiwei’ [111].

Defense lignin often differs from developmental lignin in composition and cross-linking, enhancing mechanical resistance [20]. This compositional shift can be mediated by selective activation of G-lignin biosynthetic enzymes, including HCT and C3H [20,112]. This targeted biosynthetic reprogramming is orchestrated by stress-responsive TFs. For instance, in pear, the R2R3-MYB TF *PbrMYB14* integrates structural and chemical defenses by coordinately activating lignin and SA biosynthesis pathways, enhancing resistance to *Alternaria alternata* [62]. Similarly, in herbaceous peony, *PlWRKY29* links JA and SA signaling to secondary cell wall reinforcement [50]. Emerging evidence also points to the involvement of long non-coding RNAs (lncRNAs) in regulating cell wall defense responses. In cotton, *lncRNA7* and *lncRNA2* modulate defense genes to enhance resistance against *Verticillium* wilt [113], suggesting that lncRNA-mediated regulation of defense-related cell wall modification may represent a conserved but unexplored mechanism in fruit tree-fungal pathogen interactions. Postharvest losses in tropical fruits, many of which involve lignin-related defense responses, vary widely from 10% to 80% in both developed and developing countries, underscoring the economic significance of understanding lignin-mediated disease resistance.

### 4.2. Bacterial Pathogen Resistance

This defensive lignification program is also critically deployed against bacterial pathogens. Species such as *Erwinia amylovora* (fire blight) and *Pseudomonas syringae* threaten pome fruit trees, and lignin contributes to resistance both as a preformed barrier and via inducible deposition at infection sites [20,114]. During infection, rapid lignin deposition in xylem vessels physically restricts systemic bacterial spread [20]. At the molecular level, pattern recognition receptors (PRRs) detect pathogen-associated molecular patterns (PAMPs), activating lignin biosynthetic genes via MAPK (mitogen-activated protein kinase) cascades [115,116]. This pathway is exemplified in kiwifruit (*Actinidia chinensis*) defense against *Pseudomonas syringae pv. actinidiae* (Psa), where infection triggers MAPK signaling leading to the upregulation of the lignin biosynthetic laccase gene *AcLAC35* and enhanced lignification, defining a specific transcriptional module that strengthens resistance [117]. Bacterial infection also induces accumulation of phenolic compounds, which serve as both direct antimicrobial agents and as essential precursors for the reinforcing lignin polymer deposited at infection sites [100].

ROS generated during infection serve dual functions: they directly inhibit bacterial growth and act as substrates for PRXs that catalyze monolignol polymerization, thereby facilitating rapid defense lignin formation at infection sites [20,118]. PRXs exemplify the multifunctional nature of lignin-associated enzymes, contributing both to ROS metabolism and to the oxidative coupling of monolignols [98]. This localized, rapid lignification response is a hallmark of direct pathogen confrontation. In fruit trees like apple, however, the activation of broader, systemic defense pathways—such as Systemic Acquired Resistance (SAR)—can involve distinct regulatory logic. For instance, while direct inoculation with *Erwinia amylovora* induces pathogenesis-related (PR) genes in apple, conventional chemical SAR elicitors fail to activate these genes in young shoots, underscoring unique features of systemic immunity in woody perennials [119].

### 4.3. Viral Infection and Lignification Responses

Virus infection can alter secondary metabolism, including phenylpropanoid flux that may influence lignin deposition [20,120]. Unlike fungi and bacteria, viruses frequently induce lignin deposition in vascular tissues across multiple organs, reflecting their systemic mode of infection. Lignification, together with callose accumulation at plasmodesmata and sieve tubes, restricts viral movement through the plant vascular system [20,121,122]. The plant cell wall, including lignin, functions as a dynamic defensive barrier that can be both constitutive and inducible in response to pathogen attack [20]. During viral defense, pathogen-induced ROS activate phenylpropanoid metabolism, redirecting carbon flux toward lignin and antioxidant phenolic compounds to alleviate oxidative stress [120,123].

Transcriptomic and proteomic evidence from fruit trees provides more direct support for these mechanisms. In apple, infection with apple rubbery wood virus (ARWV) reduces lignification specifically in the secondary cell walls of xylem fibers through the accumulation of virus-activated small interfering RNAs (vasiRNAs), which guide the cleavage and degradation of PAL transcripts via the RNA-induced silencing complex (RISC), thereby suppressing phenylpropanoid flux and lignin deposition. This leads to increased wood flexibility and digestibility, while mid-lamellae and xylem ray cells remain largely unaffected [124]. In contrast, infection with apple necrotic mosaic virus (ApNMV) leads to extensive changes in genes involved in secondary metabolite biosynthesis, carbohydrate metabolism, and redox regulation, with ROS accumulation closely associated with mosaic symptom development [125]. Studies in banana (*Musa paradisiaca*) further illustrate the general role of lignin in plant defense. Infection by the burrowing nematode (*Radopholus similis*) induces extensive secondary cell wall lignification in vascular bundles, while the cortex cells directly attacked by the nematode remain largely unaffected. This lignification, together with the accumulation of phenolic compounds in necrotic cells, represents an inducible defense mechanism aimed at protecting vascular tissues rather than directly preventing pathogen development in the infection site [126].

### 4.4. Insect Herbivory and Induced Lignification

Insect feeding induces rapid lignification, strengthening tissues and aiding wound healing. Mechanical damage from chewing or piercing-sucking insects triggers lignin deposition at feeding sites, reducing tissue palatability and nutritional value [127]. In fruit trees, leaf damage triggers the rapid induction of *PAL* and *CCR*, increasing lignin content to limit subsequent herbivory [127]. Herbivore-induced lignification is primarily regulated by JA signaling. JAZ repressors modulate MYB TFs controlling lignin biosynthesis, linking damage perception to defense activation [60,128]. Insect oral secretions contain elicitors that specifically trigger herbivore-responsive defense signaling and broad transcriptional reprogramming in the plant, including pathways involved in secondary metabolism and defense responses [129]. In passion (*Passiflora edulis*) fruit, insect herbivory induces local and systemic activation of lipoxygenase (LOX), a key enzyme in JA biosynthesis, confirming that the herbivore response is mediated by JA signaling [130]. Volatile organic compounds (VOCs) released during herbivory can prime neighboring tissues for systemic induced lignification, enhancing resistance [131].

In tea trees (*Camellia sinensis*), infestation by the leafhopper *Tambocerus elongatus* significantly increases lignin content and upregulates lignin biosynthetic genes, with the JA pathway playing a central role, highlighting the pivotal role of lignin in herbivore defense [132]. Rapid and extensive lignin deposition strongly reduces herbivore performance, while delayed responses may be less effective [127]. Lignification is also critical against below-ground pests. Root-parasitic nematodes, including root-knot (*Meloidogyne*) and lesion (*Pratylenchus*) nematodes, induce localized lignification at penetration sites, restricting infection. Resistant rootstocks respond with rapid lignin deposition in the cortex and endodermis, forming physical barriers that limit nematode migration and feeding site establishment [133].

**Table 2:** Lignin-mediated defense responses to biotic stresses in fruit trees.

Stress Type	Fruit Name	Key Genes/Regulators	Tissue Response	Key Findings	References
Fungal pathogens	Apple	*MdWRKY75e* (TF), *MdLAC7* (Laccase)	Leaves, infection sites	*MdWRKY75e* upregulates *MdLAC7* expression, driving defense lignification and cell wall thickening to confine *Alternaria alternata* lesions	[108]
	Apple	*MdPAL*, *Md4CL*, *MdCAD*	Leaves, infection sites	*A. alternata* infection triggers broad upregulation of core lignin genes, supporting coordinated defense lignification	[109]
	Pear	*PbrMYB14*; lignin and SA biosynthesis genes	Leaves	PbrMYB14 activates lignin and SA biosynthesis pathways, integrating structural and chemical defenses against *A. alternata*	[62]
	Litchi	*C4H*, *COMT*, *CAD*, *POD*, *SOD*, *CAT*	Fruit peel	Higher lignin accumulation and enhanced ROS scavenging in the resistant variety restrict *Peronophythora litchii* progression	[111]
Bacterial pathogens	Kiwifruit	*AcLAC35*, MAPK cascade	Infection site tissues	Psa infection triggers MAPK signaling, which upregulates *AcLAC35* and induces lignin deposition, thereby enhancing resistance	[117]
Viral infection	Apple	*PAL*, *4CL*, *CCR*, *CAD*	Xylem fibers	ARWV infection generates vasiRNAs that suppress phenylpropanoid flux, reducing SCW lignification and causing the ‘rubbery wood’ phenotype	[124]
Insect herbivory	*Camellia sinensis*	Lignin biosynthetic genes, JA pathway	Leaves	Infestation by *Tambocerus elongatus* activates the JA pathway, upregulating lignin genes and increasing lignin content for herbivore defense	[132]

Abbreviations: 4CL, 4-coumarate:CoA ligase; ARWV, apple rubbery wood virus; CAD, cinnamyl alcohol dehydrogenase; CAT, catalase; CCR, cinnamoyl-CoA reductase; COMT, caffeic acid O-methyltransferase; C4H, cinnamate 4-hydroxylase; JA, jasmonic acid; LAC, laccase; MAPK, mitogen-activated protein kinase; PAL, phenylalanine ammonia-lyase; POD, peroxidase; Psa, Pseudomonas syringae pv. actinidiae; ROS, reactive oxygen species; SA, salicylic acid; SCW, secondary cell wall; SOD, superoxide dismutase; TF, transcription factor; vasiRNA, virus-activated small interfering RNA.

## 5. Molecular Integration: Crosstalk Between Abiotic and Biotic Stress-Induced Lignification

**Figure 3:**
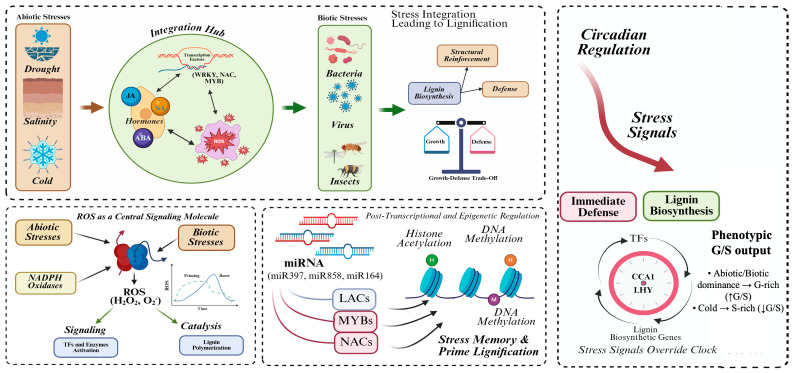
Multilayered integration of abiotic and biotic stress signaling pathways regulating lignin biosynthesis in fruit trees. This figure summarizes the molecular integration discussed in Section 5, highlighting how diverse stress signals converge through shared regulatory components. Abiotic stresses (drought, salinity, and cold) and biotic stresses (bacteria, viruses, and insects) are perceived through hormonal signaling and reactive oxygen species (ROS) accumulation, which function as central integrators. Key shared components include hormone crosstalk (jasmonic acid, JA; salicylic acid, SA; and abscisic acid, ABA), common transcription factors (WRKY, NAC, and MYB families), and ROS signaling. These signals converge to activate lignin biosynthetic programs while balancing growth–defense trade-offs. ROS acts as both signaling molecules and catalysts for lignin polymerization. Lignification is further fine-tuned by post-transcriptional regulation through microRNAs and epigenetic mechanisms (histone acetylation and DNA methylation), contributing to stress memory and primed lignification. Circadian regulation interacts with stress signaling to prioritize immediate defense, highlighting dynamic temporal control of stress-adaptive cell wall remodeling. The ultimate phenotypic output is reflected in the G/S ratio (annotated in the figure): abiotic (drought, salinity, heat) and biotic dominance drive G-rich lignin (↑ G/S), while cold favors S-rich lignin (↓ G/S).

Fruit trees in nature rarely encounter abiotic and biotic stresses in isolation, necessitating integrated defense strategies where lignification acts as a central convergence point for diverse stress signaling pathways and shares transcriptional regulators and biosynthetic machinery across stress types [134]. Rather than maintaining entirely separate networks for each stress, fruit trees co-opt developmental lignification programs to coordinate multi-stress responses. However, this robust defense entails a metabolic cost: carbon skeletons and energy invested in lignin biosynthesis are diverted from growth and reproductive processes, reflecting the classical growth–defense trade-off in plant resource allocation [135]. Understanding these crosstalk mechanisms is essential for developing climate-resilient cultivars that maintain broad-spectrum stress tolerance without compromising fruit quality. Figure 3 illustrates the multilayered integration of abiotic and biotic stress signaling pathways—including phytohormone crosstalk, ROS signaling, and epigenetic regulation—that converge on the transcriptional and post-transcriptional control of lignin biosynthesis.

### 5.1. Shared Transcriptional Regulators

Core TFs serve as integration nodes for multi-stress responses, exemplifying regulatory economy. WRKY, NAC, and MYB families mediate lignification under both abiotic and biotic stresses, allowing combinatorial control and efficient resource allocation. For instance, in pear, *PbWRKY24*—originally implicated in developmental russet formation—can be induced by osmotic stress and is associated with enhanced defense-related lignification [49]. Similarly, the apple master regulator *MdSND1* coordinates NAC-MYB cascades under salt stress and pathogen attack, linking structural reinforcement to multiple defense pathways [40].

Weighted gene co-expression network analysis (WGCNA) has begun to reveal hub genes and developmental-stage-specific connectivity in lignification regulation. In pear, transcriptomic analysis identified *PbCCR1* as a key lignin gene during early fruit development [136], while multi-omics dissection of lenticel spot formation revealed MYB TFs as central regulators of spatio-temporal lignin deposition [137]. WGCNA in pomelo also identified MYB and NAC TFs as highly correlated with lignin formation in juice sacs [138]. Collectively, these network analyses suggest that endogenous developmental signals may modulate the co-expression connectivity between hub TFs (MYB and NAC) and lignin biosynthetic modules. However, the precise regulatory logic governing lignin output—particularly how signal intensity and network topology collectively influence final lignin accumulation—remains to be functionally validated.

Phytohormones integrate these responses by modulating TF activity and metabolic flux. ABA primarily promotes drought-induced lignification via osmotic stress-responsive genes; SA activates pathogen-triggered lignification; and JA mediates wound- and herbivore-induced responses [2,139]. These hormonal pathways are integrated at multiple levels to ensure a coordinated lignification response. For instance, hormone-responsive TFs bind cis-regulatory elements in the promoters of lignin biosynthetic genes [140], hormone biosynthetic genes are co-regulated with lignin genes [15], and signaling components interact with lignification regulators to enable rapid transcriptional and metabolic responses [141]. Hormone-mediated metabolic reprogramming also modulates precursor allocation within the phenylpropanoid pathway, creating a balance between lignin production and other defense metabolites, such as flavonoids and tannins [64,142].

### 5.2. ROS as Signaling Molecules

ROS are central mediators linking abiotic and biotic stress responses to lignification. Drought, salinity, and heavy metal stress, as well as pathogen infection and herbivory, induce ROS accumulation via NADPH oxidases and other enzymatic sources [105]. Recent advances have revealed that ROS and reactive nitrogen species (RNS) are produced in a compartment-specific manner within organelles, such as chloroplasts, mitochondria, and peroxisomes, where their concentrations are tightly regulated to balance signaling functions and prevent cellular damage [143]. ROS acts both as a regulatory signal, activating lignin biosynthetic genes through oxidative stress-responsive TFs, and as catalytic agents in lignin polymerization via PODs [98]. This organelle-specific biosynthesis and spatiotemporal regulation of ROS/RNS provides a mechanistic framework for understanding how the same reactive species can direct distinct outcomes—from protective lignification under controlled conditions to oxidative damage when homeostasis is disrupted [143].

The functional outcome of ROS signaling is dictated by its spatiotemporal dynamics [105,144]. Abiotic stresses generally induce sustained, systemic ROS accumulation, promoting gradual lignification, whereas biotic stresses trigger rapid, localized bursts that initiate immediate defense lignification [118]. Cross-talk is evident when prior stress primes ROS-generating systems, enhancing responses to subsequent stresses; for example, drought-stressed plants display amplified ROS production upon pathogen challenge, accelerating lignification [3].

Post-translational modifications further refine ROS-mediated regulation. Stress-dependent phosphorylation of key enzymes, such as PAL, C4H, and COMT, adjusts catalytic activity and stability, coordinated by ROS-activated MAPKs and calcium-dependent protein kinases (CDPKs), allowing rapid modulation of lignin biosynthesis without requiring de novo transcription [7,145,146]. In fruit trees, emerging evidence supports the functional relevance of such phosphorylation cascades: in apple, *MdMAPK3*-mediated phosphorylation of the NAC TF *MdNAC72* promotes fruit softening during storage, while *MdMAPK6* phosphorylates *MdWRKY9* to regulate ripening [147]; in peach, *PpCDPK29* mediates Ca^2+^-ROS signaling and phosphorylates *PpHSFA2a* to enhance postharvest chilling tolerance, a process closely linked to lignification-related chilling injury [148]. Proteomic analyses reveal stress-specific accumulation patterns of lignin enzymes, highlighting functional specialization under combined stress conditions [19].

### 5.3. Epigenetic and miRNA-Mediated Regulation of Stress-Responsive Lignification

miRNAs and epigenetic modifications provide additional layers of regulation, integrating multi-stress lignification and establishing stress memory. Crucially, they can establish stress memory, priming the plant for faster responses to recurrent challenges. For example, miR397 targets LACs, coordinating developmental and stress-responsive lignification [149,150], while miR858 modulates MYB TFs under drought and salinity [66,151,152], and miR164 targets NAC TFs to fine-tune NAC-MYB cascades across diverse stresses [67]. Temporal expression patterns of miRNAs allow differential responses: sustained changes enable long-term metabolic adjustments under abiotic stress, while rapid, transient changes facilitate immediate defense during biotic challenges [153,154].

Epigenetic modifications establish molecular memory, priming lignification for subsequent stresses. Histone acetylation and DNA methylation dynamically respond to environmental cues, with stable CG methylation providing longer-term stress memory, while CHH/CHG methylation remains more plastic [155]. Stress-induced histone modifications and DNA methylation accelerate secondary lignification; in poplar, dissociation of *MYB94* from histone deacetylases HDA907/908 mitigates oxidative damage while enhancing subsequent lignin deposition [76]. Pathogen exposure can induce a primed state in trees, leading to stronger responses to subsequent abiotic stresses, demonstrating cross-stress epigenetic memory [16]. Interactions between miRNAs and chromatin modifications further fine-tune stress responsiveness, enabling faster lignin biosynthesis upon repeated stress [156].

While most evidence concerns within-generation memory, studies in model species suggest the potential for trans-generational inheritance of adaptive epigenetic states [157]. This represents a promising avenue for understanding long-term adaptation in perennial fruit trees, though it remains largely unexplored in the context of lignification.

### 5.4. Circadian Regulation of Lignification and Stress Integration

Circadian clocks provide temporal coordination of lignification, aligning gene expression and metabolite availability with daily rhythms. In poplar and Arabidopsis, key lignin biosynthetic genes, including *PAL*, *4CL*, and *C4H*, exhibit circadian oscillations with peak expression in the late afternoon or early evening, coinciding with maximum photosynthetic output [158,159]. Core clock components, such as circadian-clock-associated 1 (CCA1) and late elongated hypocotyl (LHY), bind promoters of secondary wall-activating MYB TFs, gating lignification in a time-of-day-dependent manner [160].

This tiered temporal regulation—baseline circadian gating overridden by acute stress signals—exemplifies the sophisticated integration that allows fruit trees to balance the metabolic cost of lignification with its essential protective benefits, coordinating defense with environmental and developmental cues. While the molecular framework for circadian gating of lignification is increasingly understood, quantitative characterization of the stress intensity threshold at which circadian control is overridden—and its variation across species and cultivars—represents a key priority for future research.

### 5.5. Growth–Defense Trade-Off: Metabolic Costs

The growth–defense trade-off imposed by lignification has quantifiable metabolic costs. Each monolignol unit requires significant ATP and NADPH investment for phenylpropanoid pathway reactions [135]. In fruit trees, this manifests as reduced soluble sugar accumulation when lignification is induced by stress [161]. In pear, GA application reduces stone cell lignification while promoting flesh expansion [14]. Quantifying these metabolic costs using isotope tracing and fluxomics represents a key frontier for optimizing the growth–defense balance in fruit production.

### 5.6. Combined Stress Interactions and Lignin Reprogramming

In natural environments, fruit trees frequently encounter combined abiotic and biotic stresses, which can elicit lignin regulatory outcomes distinct from individual stress responses [3,13,105]. This complexity arises from crosstalk between shared transcription factors (WRKY, NAC, and MYB), overlapping hormonal networks (ABA, JA, and SA), and altered ROS dynamics [40,49,108]. For example, drought priming can accelerate pathogen-induced lignification, while prolonged drought may suppress JA-mediated defense responses by diverting carbon flux toward osmotic adjustment [3,13]. Cold-induced lignification can also compromise pathogen resistance through metabolic reallocation [90,94]. Such non-linear interactions depend on stress severity, timing, and sequence, challenging predictions under climate change. Future research must move beyond single-stress paradigms to identify hub genes maintaining lignin homeostasis across diverse stress combinations.

## 6. Developmental and Postharvest Dynamics of Lignification: Implications for Fruit Quality

**Figure 4:**
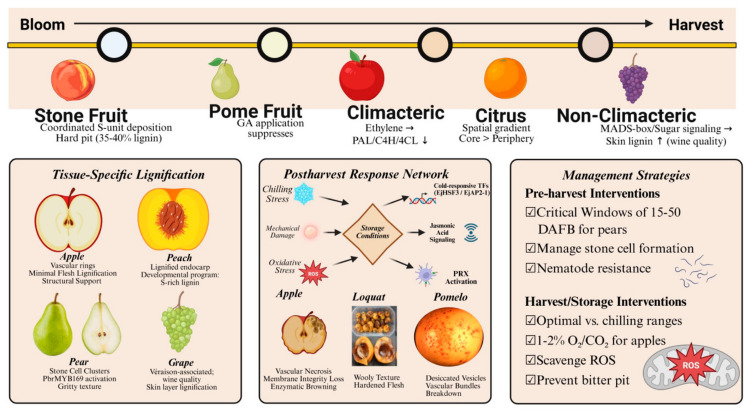
Developmental and postharvest dynamics of lignification in fruit trees. Timeline of developmental lignification illustrating species-specific patterns and regulatory windows: stone fruits exhibit coordinated S-unit deposition (35–40% lignin) in the endocarp; pome fruits show gibberellic acid (GA)-sensitive lignification suppression; climacteric fruits undergo ethylene-mediated downregulation of lignin biosynthetic genes (*PAL*, *C4H*, and *4CL*); citrus displays spatial lignification gradients from core to periphery; and non-climacteric fruits rely on MADS-box and sugar signaling to regulate skin lignification. Tissue-specific localization distinguishes structural lignification (peach endocarp and apple vascular rings) from quality-affecting lignification (pear stone cells and grape skin at véraison). Postharvest stress-responsive networks integrate chilling, mechanical damage, and oxidative stress through transcriptional and enzymatic regulation, leading to lignin-associated disorders, such as wooliness, hardened flesh, vascular necrosis, and juice sac granulation. Management strategies target critical developmental windows and storage conditions to balance lignification with fruit quality.

Lignification in fruit trees is a dynamic, spatially and temporally regulated process that occurs throughout fruit development and continues to respond to environmental cues during postharvest storage. Understanding these patterns is critical for optimizing fruit quality and informing management strategies. Figure 4 illustrates the developmental and postharvest dynamics of lignification in fruit trees, highlighting temporal changes, regulatory mechanisms, and their implications for fruit quality. Table 3, in turn, summarizes specific lignification patterns and their regulatory mechanisms across different fruits, organized by developmental stage or storage condition, quality impact, and references.

### 6.1. Lignification During Fruit Development and Ripening

Lignification patterns during fruit development vary among species, reflecting tissue-specific regulatory programs and metabolic priorities. These patterns can manifest as progressive, developmentally programmed accumulation. For example, in pomelo, juice sac granulation is accompanied by progressive lignin accumulation after anthesis, with lignin content increasing from early to late developmental stages and remaining consistently higher in juice sacs near the fruit core than in those farther from the core, indicating spatial regulation of lignification during granulation [138]. Similarly, in winter jujube (*Ziziphus jujuba*), pericarp lignin content steadily increases during pigmentation, indicating that lignification accompanies pigment accumulation and cell wall modification [18]. In oil-tea camellia (*Camellia oleifera*), lignin progressively accumulates in stone cell walls during shell growth, thickening cell walls and likely enhancing mechanical protection of seeds [162].

Beyond generalized tissue reinforcement, some species exhibit highly programmed developmental lignification. Stone fruits are a prime example; in peaches, endocarp lignification begins approximately 25–30 days after full bloom, continuing for 2–3 weeks. This process involves coordinated upregulation of lignin biosynthetic genes and TFs that activate endocarp-specific programs, resulting in mature stones containing 35–40% lignin with high syringyl content, contributing to stone hardness [10]. In pome fruits, however, similar lignification processes can negatively impact quality. In pears, stone cells develop early in fruit growth (15–50 days after bloom), contributing to a stone cell gritty texture. *PbrMYB169* promotes this process by activating lignin biosynthetic genes such as *PbrPAL1*, *Pbr4CL1*, and *PbrCAD1* [163]. GA signaling is associated with the suppression of stone cell formation, indicating that manipulating GA pathways during key developmental windows may reduce stone cell content [14]. Pear, with an annual worldwide production of approximately 24 million tons, ranks among the most economically important temperate fruit crops. The presence of stone cells directly reduces fruit eating quality, consumer satisfaction, and export value, making them explicit targets for reduction in pear breeding programs.

In apple fruit flesh, lignification is typically minimal under normal conditions, being largely confined to xylem cells within vascular bundles for structural support [164]. However, postharvest abiotic stress, such as photooxidative stress, can activate phenylpropanoid and ethylene pathways, inducing widespread ectopic lignification throughout the flesh parenchyma and altering fruit texture [165]. This contrast—from developmentally confined, functionally beneficial lignification to stress-induced, quality-compromising ectopic deposition—exemplifies the central ‘dual role’ thesis of this review (see Figure 4), highlighting how the same lignification machinery, when dysregulated by postharvest stress, shifts from structural maintenance to textural deterioration.

The regulatory control of lignification undergoes a major shift during the transition from development to ripening, with fundamental differences between climacteric and non-climacteric fruit types. Transcriptomic analyses of apple fruit ripening indicate broader shifts in phenylpropanoid-related gene expression during this phase, consistent with a reduced capacity for lignin biosynthesis in the flesh [166]. Notably, vascular tissues maintain their lignification potential throughout ripening to preserve structural integrity [167]. Conversely, in non-climacteric fruits like grapes, skin phenolic composition changes significantly during véraison (the onset of fruit ripening) and continues through maturation, contributing to traits critical for wine quality, such as astringency and color. These processes are influenced by developmental cues and hormonal regulation, including ABA signaling [168,169].

Beyond their role in fresh fruit quality, lignified tissues from processed fruit represent a substantial biomass resource. Apple pomace—comprising peels, cores, seeds, and residual flesh—contains substantial structural polymers, including cellulose, hemicellulose, and lignin, often reported at ~20–30% depending on fraction and processing [170]. Understanding these tissue- and species-specific regulatory programs, from development through ripening, is essential for optimizing both texture and sensory quality in fresh fruit while harnessing byproduct value. Notably, the postharvest environment itself can be actively managed to steer other key quality parameters. Beyond structural lignification, light quality—particularly UV radiation—serves as a key postharvest signal to regulate ripening-related pigmentation, a major determinant of visual quality and marketability. For instance, postharvest UV-A treatment of mango (*Mangifera indica*) fruit upregulates light-signaling (*MiHY5*) and pigment biosynthesis genes (*MiMYB1* and *MiANS*), promoting anthocyanin and carotenoid accumulation in the peel while accelerating ripening processes, such as cell wall degradation [171]. This demonstrates that targeted light treatments can be used as a practical tool to decouple and optimize specific quality traits like color during postharvest handling, complementing strategies aimed at controlling textural changes via lignification.

### 6.2. Postharvest Lignification and Storage Disorders

Postharvest lignification presents major quality concerns, as progressive cell wall modifications during storage can compromise texture, palatability, and market value. Oxidative stress, temperature fluctuations, and mechanical damage activate lignin biosynthesis, accelerating quality deterioration. Chilling injury is a primary trigger, particularly in tropical and subtropical fruits. In loquat, storage at 0 °C rapidly induces lignin biosynthetic genes (*EjPAL1*, *Ej4CL1*, and *EjCAD1*), resulting in flesh lignification and woolly texture within 7–14 days [172]. Cold-responsive TFs, including *EjHSF3* and *EjAP2-1*, regulate this process: *EjHSF3* activates lignin biosynthesis, whereas *EjAP2-1* represses it via interaction with EjMYB TFs [11,173,174]. In pear, cold storage-induced lenticel disorder is a direct consequence of ectopic lignification. The expansion and protrusion of lenticels are driven by the upregulation of lignin biosynthetic genes associated with ethylene- and jasmonate-related signaling pathways, providing a clear case where hormone crosstalk exacerbates a postharvest lignification disorder [175]. Cold stress may disrupt this repression via the degradation or sequestration of the repressor *EjAP2-1*, thereby derepressing the lignin biosynthetic pathway and triggering lignification [176]. Chilling-induced ROS accumulation further promotes lignin polymerization.

In mango, cultivar-specific susceptibility to chilling injury correlates with differences in cell wall integrity and membrane stability. For example, ‘Langra’ exhibits only 5% chilling injury compared to 36% in ‘Gulab Jamun’ after 28 days at 5 °C [177]. Chilling injury leads to decreased marketability and substantial food waste in mango and other tropical fruits. Chilling injury alone accounts for more than one-third of total postharvest economic losses for tropical and subtropical products.

In pomelo, postharvest lignification is associated with juice sac granulation, elevated PAL and PRX activity, sucrose degradation, and disrupted energy metabolism, linking lignification to broader metabolic deterioration [161]. Controlled-atmosphere storage and ethylene-modulating treatments primarily alleviate postharvest physiological disorders by suppressing ethylene action and reducing oxidative stress; for example, apples stored under low O_2_ and CO_2_ conditions exhibit a reduced incidence of core browning compared with fruit held under regular atmosphere conditions [178]. Because oxidative stress and ethylene signaling are key drivers of stress-induced phenylpropanoid activation, these storage strategies likely create conditions that are less permissive for postharvest lignification. Proper temperature management and careful handling are therefore critical, as mechanical injury can induce JA-associated lignification responses, and physiological disorders, such as core browning or bitter pit, are closely linked to oxygen stress and calcium deficiency.

### 6.3. Implications for Fruit Quality and General Management Insights

Understanding lignification dynamics provides direct strategies for managing fruit quality from orchard to market. Effective intervention requires integrating developmental timing, environmental monitoring, and stress mitigation. Pre-harvest, applying plant growth regulators during key developmental windows can suppress undesirable lignification to improve fresh fruit texture at harvest. Postharvest, management should focus on modulating the triggers of stress-induced lignification. This includes optimizing storage temperature and atmosphere to minimize chilling injury, using antioxidant treatments to scavenge ROS, and applying targeted hormone treatments to maintain a repressive state in lignification pathways. Implementing these practices, informed by the molecular insights reviewed here, forms a practical framework for preserving texture, palatability, and market value across diverse fruit species.

**Table 3:** Developmental and postharvest lignification patterns in fruit trees and their quality implications.

Fruit Type	Fruit Name	Developmental Stage/Storage Condition	Lignification Pattern & Regulation	Quality Impact	References
Citrus	Pomelo	Post-anthesis fruit development	Lignin accumulates progressively after anthesis, with higher deposition near the core, indicating spatial regulation of juice sac lignification via differential PAL and PRX activity	Juice sac granulation	[138]
	Pomelo	Postharvest storage	Lignin accumulation is associated with enhanced PAL and PRX activity, sucrose degradation, and disrupted energy metabolism	Juice sac granulation; metabolic deterioration	[161]
Stone fruits	Peach	Stone development –lignification initiation (25–30 DAFB; continues 2–3 weeks)	Coordinated endocarp lignification regulated by lignin biosynthetic genes and endocarp-specific TFs; mature stones contain 35–40% lignin with high syringyl content	Stone hardness (desirable trait)	[10]
Pome fruits	Pear	Early fruit growth (15–50 DAFB)	*PbrMYB169* upregulates *PbrPAL1*, *Pbr4CL1*, and *PbrCAD1* expression to drive stone cell development; GA signaling suppresses this process	Stone cell gritty texture (undesirable); mitigated by strategic GA application during critical developmental windows	[14,163]
	Pear	Domestication/ripening	Global increases in DNA methylation correlate with downregulation of phenylpropanoid biosynthesis genes, contributing to early fruit ripening	Altered ripening timing; reduced lignin-related gene expression	[74]
	Pear	Cold storage	Cold storage triggers ethylene- and JA-mediated upregulation of lignin genes, leading to lignified and protruding lenticels	Surface roughness, reduced marketability (storage disorder)	[175]
	Apple	On-tree development (40 DAFB to harvest)	Lignification is developmentally programmed and confined to xylem cells, providing structural integrity without affecting flesh quality	Maintains vascular function and edible, non-lignified flesh	[164]
	Apple	Postharvest abiotic stress (photooxidative: high light exposure combined with wounding)	Stress activates ethylene and phenylpropanoid pathways, revealing high lignification potential in parenchyma cells	Undesirable textural hardening (loss of crispness/mealiness)	[165]
Tropical fruits	Loquat	Postharvest storage at 0 °C (7–14 days)	Chilling induces rapid flesh lignification via upregulation of *EjPAL1*, *Ej4CL1*, and *EjCAD1*; *EjHSF3* activates while *EjAP2-1* represses the pathway; ROS promotes polymerization. Additionally, EjNAC5 promoter methylation is negatively correlated with its transcript abundance, and lower methylation in chilling-susceptible cultivars drives lignification	Woolly texture; chilling injury	[11,75,172,173,174,176]
Other	Winter jujube	Fruit pigmentation period	Pericarp lignin content continuously increases, coordinated with pigment accumulation	Cell wall modification during fruit maturation	[18]
	Oil-tea camellia	Fruit growth	Lignin progressively accumulates in the fruit shell, thickening stone cell walls and reinforcing structural integrity	Mechanical protection of seeds	[162]

Abbreviations: 4CL, 4-coumarate:CoA ligase; AP2, APETALA2 transcription factor; CAD, cinnamyl alcohol dehydrogenase; DAFB, days after full bloom; GA, gibberellic acid; HSF, heat shock factor; JA, jasmonic acid; PAL, phenylalanine ammonia-lyase; PRX, peroxidase; ROS, reactive oxygen species; TF, transcription factor.

## 7. Summary and Future Perspectives

Lignification in fruit trees encapsulates a central physiological and agronomic trade-off, balancing indispensable structural defense against the imperative for high fruit quality. This equilibrium is governed by sophisticated, multilayered regulatory networks. The conserved NAC–MYB transcriptional module acts as the core integrator, coordinating with WRKY/ERF transcription factors (TFs), microRNA-guided post-transcriptional regulation, and epigenetic modifications that establish stress memory. Under abiotic stress, targeted lignification reinforces vasculature, limits ion flux, and mitigates thermal and oxidative damage, whereas rapid defense lignification forms physical barriers against pathogens and herbivores. Extensive molecular crosstalk through shared TFs, interconnected phytohormone signaling, and reactive oxygen species (ROS) enables coordinated responses to multiple environmental challenges. Conversely, dysregulation of these same pathways contributes to undesirable fruit lignification and postharvest textural disorders, emphasizing the need for precise spatiotemporal regulation that enhances stress resilience without compromising fruit quality. Despite substantial progress, several critical knowledge gaps continue to limit the translation of lignification research into practical horticultural applications. Future research should prioritize the following three complementary directions.

### 7.1. Tissue-Specific Genome Editing for Functional Validation of Lignification Regulators

CRISPR/Cas9 has demonstrated considerable promise for investigating lignin biosynthesis in fruit trees. In pear (*Pyrus* spp.), highly efficient CRISPR/Cas9 and CRISPRa systems have been established in calli, achieving near 100% multiplex editing efficiency [179]. Similarly, knockout of *MdLAC7* and *MdCCR1* in apple (*Malus domestica*) has confirmed their essential roles in lignin biosynthesis, while *MdMYB88* and *MdMYB124* have been identified as promising targets for improving stress tolerance through lignification [72,180]. However, current functional validation remains largely confined to callus tissues or early-stage transformation systems, and efficient tissue-specific genome editing in intact fruit trees is still lacking. Future studies should prioritize the development of tissue-specific promoters together with multiplex CRISPR/Cas, base-editing, and prime-editing strategies to manipulate NAC–MYB regulatory networks in specific lignifying tissues, such as pear stone cells, peach endocarp, vascular tissues, and fruit peel. Such approaches would enable precise modification of stress-responsive lignification while minimizing undesirable effects on whole-plant growth and fruit quality.

### 7.2. Single-Cell and Single-Nucleus Omics to Resolve Cell-Specific Lignification Programs

Recent advances in single-cell transcriptomics have begun to uncover the cellular complexity of lignification. In pitaya (*Selenicereus undatus*), single-cell RNA sequencing revealed cell-type-specific phenylpropanoid branching, where *HuMYBS3* differentially regulates lignin (*HuCOMT1*) and flavonoid (*HuCHI*) biosynthesis in distinct exocarp cell layers [181]. Integrated multi-omics has also elucidated endocarp lignification networks in walnut (*Juglans regia*) [182], while single-nucleus RNA-seq atlases are becoming available for apple [183]. Nevertheless, direct application of single-cell or single-nucleus transcriptomics to lignifying tissues in fruit trees remains extremely limited. In particular, the cellular heterogeneity underlying pear stone cell formation and peach (*Prunus persica*) endocarp lignification remains poorly understood because most available datasets rely on bulk transcriptomic analyses. Furthermore, technical challenges associated with protoplast isolation from highly lignified woody tissues, low cell recovery, and limited spatial resolution continue to restrict widespread application of these technologies. Future integration of single-cell, single-nucleus, spatial transcriptomics, and spatial metabolomics will enable unprecedented resolution of cell-type-specific regulatory networks governing lignification and facilitate precision breeding for improved fruit quality.

### 7.3. Quantitative Fluxomics to Characterize Growth–Defense Metabolic Trade-Offs

Lignification imposes substantial metabolic costs because monolignol biosynthesis requires considerable adenosine triphosphate (ATP), nicotinamide adenine dinucleotide phosphate (NADPH), and carbon allocation from primary metabolism. Despite extensive transcriptomic and metabolomic studies, quantitative understanding of carbon flux into lignin remains remarkably limited in fruit trees. Current studies primarily describe differential gene expression or metabolite accumulation but rarely quantify how carbon partitioning changes during stress-induced lignification or how these metabolic shifts affect fruit growth and quality. Future research should integrate stable isotope labeling (e.g., ^13^C), metabolic fluxomics, transcriptomics, metabolomics, and physiological analyses to quantify carbon allocation among lignin, sugars, organic acids, and other phenylpropanoid-derived metabolites. Such quantitative approaches will provide mechanistic insight into the growth–defense trade-off and identify metabolic thresholds that maximize stress tolerance while minimizing adverse effects on fruit quality.

Beyond these priorities, complementary approaches—including digital agriculture, hyperspectral imaging, artificial intelligence-assisted phenotyping, genome-wide association studies, microbiome-assisted stress priming, and the valorization of lignin-rich processing byproducts—will further accelerate the development of climate-resilient fruit production systems. Integrating these emerging technologies with precision genome editing and systems-level multi-omics will facilitate the breeding of cultivars capable of maintaining both environmental resilience and superior fruit quality under increasingly variable climatic conditions.

In conclusion, lignin metabolism is not a static endpoint but a dynamic, information-rich process central to the perennial fruit tree’s ability to balance growth, defense, and reproduction. Addressing the three key research priorities outlined above—tissue-specific genome editing, cell-resolved lignification analysis, and quantitative metabolic flux characterization—will substantially advance our mechanistic understanding of lignification and accelerate the development of sustainable, climate-resilient fruit production systems without sacrificing the sensory quality that defines consumer acceptance.

## Data Availability

All data supporting the findings of this review are included in the article. Further inquiries can be directed to the corresponding author.

## Supplementary Materials

The following supporting information can be downloaded at https://www.mdpi.com/article/10.3390/plants15142244/s1. Table S1 provides a complete list of abbreviations. Table S2 provides reported lignin-related genes across fruit tree species.

## References

[1] Roussos P.A. (2024). Climate change challenges in temperate and sub-tropical fruit tree cultivation. Encyclopedia.

[2] Moura J.C.M.S., Bonine C.A.V., de Oliveira Fernandes Viana J., Dornelas M.C., Mazzafera P. (2010). Abiotic and biotic stresses and changes in the lignin content and composition in plants. J. Integr. Plant Biol..

[3] Atkinson N.J., Urwin P.E. (2012). The interaction of plant biotic and abiotic stresses: From genes to the field. J. Exp. Bot..

[4] Boerjan W., Ralph J., Baucher M. (2003). Lignin biosynthesis. Annu. Rev. Plant Biol..

[5] Bonawitz N.D., Chapple C. (2010). The genetics of lignin biosynthesis: Connecting genotype to phenotype. Annu. Rev. Genet..

[6] Vanholme R., Demedts B., Morreel K., Ralph J., Boerjan W. (2010). Lignin biosynthesis and structure. Plant Physiol..

[7] Barros J., Serk H., Granlund I., Pesquet E. (2015). The cell biology of lignification in higher plants. Ann. Bot..

[8] Brodersen C.R., McElrone A.J. (2013). Maintenance of xylem network transport capacity: A review of embolism repair in vascular plants. Front. Plant Sci..

[9] Cai Y., Li G., Nie J., Lin Y., Nie F., Zhang J., Xu Y. (2010). Study of the structure and biosynthetic pathway of lignin in stone cells of pear. Sci. Hortic..

[10] Dardick C.D., Callahan A.M., Chiozzotto R., Schaffer R.J., Piagnani M.C., Scorza R. (2010). Stone formation in peach fruit exhibits spatial coordination of the lignin and flavonoid pathways and similarity to Arabidopsis dehiscence. BMC Biol..

[11] Xu Q., Yin X.-R., Zeng J.-K., Ge H., Song M., Xu C.-J., Li X., Ferguson I.B., Chen K.-S. (2014). Activator-and repressor-type MYB transcription factors are involved in chilling injury induced flesh lignification in loquat via their interactions with the phenylpropanoid pathway. J. Exp. Bot..

[12] Zhong R., Ye Z.-H. (2015). Secondary cell walls: Biosynthesis, patterned deposition and transcriptional regulation. Plant Cell Physiol..

[13] Li X., Ma Z., Song Y., Shen W., Yue Q., Khan A., Tahir M.M., Wang X., Malnoy M., Ma F. (2023). Insights into the molecular mechanisms underlying responses of apple trees to abiotic stresses. Hortic. Res..

[14] Cao Y., Li X., Jiang L. (2019). Integrative analysis of the core fruit lignification toolbox in pear reveals targets for fruit quality bioengineering. Biomolecules.

[15] Zhang J., Tuskan G.A., Tschaplinski T.J., Muchero W., Chen J.-G. (2020). Transcriptional and post-transcriptional regulation of lignin biosynthesis pathway genes in *Populus*. Front. Plant Sci..

[16] Ma H., Su L., Zhang W., Sun Y., Li D., Li S., Lin Y.C.J., Zhou C., Li W. (2025). Epigenetic regulation of lignin biosynthesis in wood formation. New Phytol..

[17] Zhao Q. (2016). Lignification: Flexibility, biosynthesis and regulation. Trends Plant Sci..

[18] Zhang Q., Wang L., Wang Z., Zhang R., Liu P., Liu M., Liu Z., Zhao Z., Wang L., Chen X. (2021). The regulation of cell wall lignification and lignin biosynthesis during pigmentation of winter jujube. Hortic. Res..

[19] Liu Q., Luo L., Zheng L. (2018). Lignins: Biosynthesis and biological functions in plants. Int. J. Mol. Sci..

[20] Miedes E., Vanholme R., Boerjan W., Molina A. (2014). The role of the secondary cell wall in plant resistance to pathogens. Front. Plant Sci..

[21] Humphreys J.M., Chapple C. (2002). Rewriting the lignin roadmap. Curr. Opin. Plant Biol..

[22] Xu B., Escamilla-Treviño L.L., Sathitsuksanoh N., Shen Z., Shen H., Percival Zhang Y.H., Dixon R.A., Zhao B. (2011). Silencing of 4-coumarate: Coenzyme A ligase in switchgrass leads to reduced lignin content and improved fermentable sugar yields for biofuel production. New Phytol..

[23] Vanholme R., Morreel K., Darrah C., Oyarce P., Grabber J.H., Ralph J., Boerjan W. (2012). Metabolic engineering of novel lignin in biomass crops. New Phytol..

[24] Hamberger B., Bak S. (2013). Plant P450s as versatile drivers for evolution of species-specific chemical diversity. Philos. Trans. R. Soc. B Biol. Sci..

[25] Eudes A., Sathitsuksanoh N., Baidoo E.E., George A., Liang Y., Yang F., Singh S., Keasling J.D., Simmons B.A., Loqué D. (2015). Expression of a bacterial 3-dehydroshikimate dehydratase reduces lignin content and improves biomass saccharification efficiency. Plant Biotechnol. J..

[26] Alejandro S., Lee Y., Tohge T., Sudre D., Osorio S., Park J., Bovet L., Lee Y., Geldner N., Fernie A.R. (2012). AtABCG29 is a monolignol transporter involved in lignin biosynthesis. Curr. Biol..

[27] Li J., Liu X., Cao Z., Yu Q., Li M., Qin G. (2025). Pomegranate ATP-binding cassette transporter PgABCG9 plays a negative regulatory role in lignin accumulation. Int. J. Biol. Macromol..

[28] Zhou M.-M., Yu Z.-H., Gao H.-N., Li M.-R., Wu Y.-T., Li H.-Y., Wang T., Lv Y.-H., Jiang H., Li Y.-Y. (2023). Ectopic expression of an apple ABCG transporter gene *MdABCG25* increases plant cuticle wax accumulation and abiotic stress tolerance. Fruit Res..

[29] Yang Z., Lin M., Yang X., Wu D., Chen K. (2023). Comprehensive analysis of transcriptome and metabolome provides insights into the stress response mechanisms of apple fruit to postharvest impact damage. Food Chem. Mol. Sci..

[30] Hou Z., Jia B., Li F., Liu P., Liu L., Ye Z., Zhu L., Wang Q., Heng W. (2018). Characterization and expression of the ABC family (G group) in ‘Dangshansuli’pear (*Pyrus bretschneideri* Rehd.) and its russet mutant. Genet. Mol. Biol..

[31] Le Roy J., Huss B., Creach A., Hawkins S., Neutelings G. (2016). Glycosylation is a major regulator of phenylpropanoid availability and biological activity in plants. Front. Plant Sci..

[32] Wang H., Zhang Y., Feng X., Peng F., Mazoor M.A., Zhang Y., Zhao Y., Han W., Lu J., Cao Y. (2022). Analysis of the β-glucosidase family reveals genes involved in the lignification of stone cells in Chinese white pear (*Pyrus bretschneideri* Rehd.). Front. Plant Sci..

[33] Speeckaert N., Adamou N.M., Hassane H.A., Baldacci-Cresp F., Mol A., Goeminne G., Boerjan W., Duez P., Hawkins S., Neutelings G. (2020). Characterization of the UDP-glycosyltransferase UGT72 family in poplar and identification of genes involved in the glycosylation of monolignols. Int. J. Mol. Sci..

[34] Amadou Hassane H., Behr M., Guérin C., Sibout R., Mol A., Baragé M., El Jaziri M., Baucher M. (2022). A higher lignin content in *ugt72b37* poplar mutants indicates a role of monolignol glycosylation in xylem lignification. Forests.

[35] Zhao Q., Nakashima J., Chen F., Yin Y., Fu C., Yun J., Shao H., Wang X., Wang Z.-Y., Dixon R.A. (2013). Laccase is necessary and nonredundant with peroxidase for lignin polymerization during vascular development in *Arabidopsis*. Plant Cell.

[36] Marjamaa K., Kukkola E.M., Fagerstedt K.V. (2009). The role of xylem class III peroxidases in lignification. J. Exp. Bot..

[37] Kärkönen A., Kuchitsu K. (2015). Reactive oxygen species in cell wall metabolism and development in plants. Phytochemistry.

[38] Nakano Y., Yamaguchi M., Endo H., Rejab N.A., Ohtani M. (2015). NAC-MYB-based transcriptional regulation of secondary cell wall biosynthesis in land plants. Front. Plant Sci..

[39] Duan R., Zhang X., Liu Y., Wang L., Yang J., Wang L., Wang S., Su Y., Xue H. (2023). Transcriptome and Physiological Analysis Highlight Lignin Metabolism of the Fruit Dots Disordering during Postharvest Cold Storage in ‘Danxiahong’ Pear. Genes.

[40] Chen K., Guo Y., Song M., Liu L., Xue H., Dai H., Zhang Z. (2020). Dual role of *MdSND1* in the biosynthesis of lignin and in signal transduction in response to salt and osmotic stress in apple. Hortic. Res..

[41] Zhong R., Richardson E.A., Ye Z.-H. (2007). Two NAC domain transcription factors, SND1 and NST1, function redundantly in regulation of secondary wall synthesis in fibers of *Arabidopsis*. Planta.

[42] Chen K., Song M., Guo Y., Liu L., Xue H., Dai H., Zhang Z. (2019). Md MYB 46 could enhance salt and osmotic stress tolerance in apple by directly activating stress-responsive signals. Plant Biotechnol. J..

[43] Li X., Wang N., She W., Guo Z., Pan H., Yu Y., Ye J., Pan D., Pan T. (2022). Identification and Functional Analysis of the *CgNAC043* Gene Involved in Lignin Synthesis from *Citrus grandis* “San Hong”. Plants.

[44] Wang R., Xue Y., Fan J., Yao J.-L., Qin M., Lin T., Lian Q., Zhang M., Li X., Li J. (2021). A systems genetics approach reveals PbrNSC as a regulator of lignin and cellulose biosynthesis in stone cells of pear fruit. Genome Biol..

[45] Xue Y., Shan Y., Yao J.-L., Wang R., Xu S., Liu D., Ye Z., Lin J., Li X., Xue C. (2023). The transcription factor PbrMYB24 regulates lignin and cellulose biosynthesis in stone cells of pear fruits. Plant Physiol..

[46] Gong X., Qi K., Zhao L., Xie Z., Pan J., Yan X., Shiratake K., Zhang S., Tao S. (2024). PbAGL7–PbNAC47–PbMYB73 complex coordinately regulates PbC3H1 and PbHCT17 to promote the lignin biosynthesis in stone cells of pear fruit. Plant J..

[47] Zhou J., Lee C., Zhong R., Ye Z.-H. (2009). MYB58 and MYB63 are transcriptional activators of the lignin biosynthetic pathway during secondary cell wall formation in *Arabidopsis*. Plant Cell.

[48] Legay S., Lacombe E., Goicoechea M., Briere C., Séguin A., Mackay J., Grima-Pettenati J. (2007). Molecular characterization of EgMYB1, a putative transcriptional repressor of the lignin biosynthetic pathway. Plant Sci..

[49] Wang J., Wang D., Zhao M., Yu M., Zheng X., Tian Y., Sun Z., Liu X., Wang C., Ma C. (2025). A transcription factor, PbWRKY24, contributes to russet skin formation in pear fruits by modulating lignin accumulation. Hortic. Res..

[50] Wu Y., Dong Z., Yin Y., Tao J., Zhao D., Tang Y. (2025). Regulation of lignin biosynthesis by a group IIe WRKY transcription factor PlWRKY29 in herbaceous peony. J. Integr. Agric..

[51] Guillaumie S., Mzid R., Méchin V., Léon C., Hichri I., Destrac-Irvine A., Trossat-Magnin C., Delrot S., Lauvergeat V. (2010). The grapevine transcription factor WRKY2 influences the lignin pathway and xylem development in tobacco. Plant Mol. Biol..

[52] Liu Y., Liu Q., Li X., Zhang Z., Ai S., Liu C., Ma F., Li C. (2023). MdERF114 enhances the resistance of apple roots to *Fusarium solani* by regulating the transcription of MdPRX63. Plant Physiol..

[53] Park S.-Y., Fung P., Nishimura N., Jensen D.R., Fujii H., Zhao Y., Lumba S., Santiago J., Rodrigues A., Chow T.-F.F. (2009). Abscisic acid inhibits type 2C protein phosphatases via the PYR/PYL family of START proteins. Science.

[54] Sheard L.B., Tan X., Mao H., Withers J., Ben-Nissan G., Hinds T.R., Kobayashi Y., Hsu F.-F., Sharon M., Browse J. (2010). Jasmonate perception by inositol-phosphate-potentiated COI1–JAZ co-receptor. Nature.

[55] Wang Z.-Y., Bai M.-Y., Oh E., Zhu J.-Y. (2012). Brassinosteroid signaling network and regulation of photomorphogenesis. Annu. Rev. Genet..

[56] Ye H., Li L., Guo H., Yin Y. (2012). MYBL2 is a substrate of GSK3-like kinase BIN2 and acts as a corepressor of BES1 in brassinosteroid signaling pathway in *Arabidopsis*. Proc. Natl. Acad. Sci. USA.

[57] Sun Y., Fan X.-Y., Cao D.-M., Tang W., He K., Zhu J.-Y., He J.-X., Bai M.-Y., Zhu S., Oh E. (2010). Integration of brassinosteroid signal transduction with the transcription network for plant growth regulation in *Arabidopsis*. Dev. Cell.

[58] Davière J.-M., Achard P. (2013). Gibberellin signaling in plants. Development.

[59] Cutler S.R., Rodriguez P.L., Finkelstein R.R., Abrams S.R. (2010). Abscisic acid: Emergence of a core signaling network. Annu. Rev. Plant Biol..

[60] Kazan K., Manners J.M. (2013). MYC2: The master in action. Mol. Plant.

[61] Liu T., Luo T., Guo X., Zou X., Zhou D., Afrin S., Li G., Zhang Y., Zhang R., Luo Z. (2019). PgMYB2, a MeJA-responsive transcription factor, positively regulates the dammarenediol synthase gene expression in *Panax ginseng*. Int. J. Mol. Sci..

[62] Yan Q., Chen W., Zhang H., Liu P., Zhang Y. (2025). PbrMYB14 enhances pear resistance to alternaria alternata by regulating genes in lignin and salicylic acid biosynthesis pathways. Int. J. Mol. Sci..

[63] Zhong R., Ye Z.-H. (2014). Complexity of the transcriptional network controlling secondary wall biosynthesis. Plant Sci..

[64] Dong N.Q., Lin H.X. (2021). Contribution of phenylpropanoid metabolism to plant development and plant–environment interactions. J. Integr. Plant Biol..

[65] Dong C.-H., Pei H. (2014). Over-expression of miR397 improves plant tolerance to cold stress in *Arabidopsis thaliana*. J. Plant Biol..

[66] Sharma D., Tiwari M., Pandey A., Bhatia C., Sharma A., Trivedi P.K. (2016). MicroRNA858 is a potential regulator of phenylpropanoid pathway and plant development. Plant Physiol..

[67] Kim J.H., Woo H.R., Kim J., Lim P.O., Lee I.C., Choi S.H., Hwang D., Nam H.G. (2009). Trifurcate feed-forward regulation of age-dependent cell death involving miR164 in *Arabidopsis*. Science.

[68] Shi R., Sun Y.-H., Li Q., Heber S., Sederoff R., Chiang V.L. (2010). Towards a systems approach for lignin biosynthesis in *Populus trichocarpa*: Transcript abundance and specificity of the monolignol biosynthetic genes. Plant Cell Physiol..

[69] Lavhale S.G., Kalunke R.M., Giri A.P. (2018). Structural, functional and evolutionary diversity of 4-coumarate-CoA ligase in plants. Planta.

[70] Nie T., Sun X., Wang S., Wang D., Ren Y., Chen Q. (2023). Genome-wide identification and expression analysis of the 4-coumarate: CoA ligase gene family in *Solanum tuberosum*. Int. J. Mol. Sci..

[71] Duplan V., Rivas S. (2014). E3 ubiquitin-ligases and their target proteins during the regulation of plant innate immunity. Front. Plant Sci..

[72] Geng D., Shen X., Xie Y., Yang Y., Bian R., Gao Y., Li P., Sun L., Feng H., Ma F. (2020). Regulation of phenylpropanoid biosynthesis by MdMYB88 and MdMYB124 contributes to pathogen and drought resistance in apple. Hortic. Res..

[73] Hemm M.R., Rider S.D., Ogas J., Murry D.J., Chapple C. (2004). Light induces phenylpropanoid metabolism in *Arabidopsis* roots. Plant J..

[74] Song B., Yu J., Li X., Li J., Fan J., Liu H., Wei W., Zhang L., Gu K., Liu D. (2024). Increased DNA methylation contributes to the early ripening of pear fruits during domestication and improvement. Genome Biol..

[75] Huang Y., Liang Z., Lu J., Zhang M., Cao X., Hu R., Li D., Grierson D., Chen W., Zhu C. (2024). The transcription factor EjNAC5 regulates loquat fruit chilling lignification. J. Exp. Bot..

[76] Kong X., Chen Y., Li H., Li M., Liu X., Xia L., Zhang S. (2024). Dissociation of transcription factor MYB94 and histone deacetylases HDA907/908 alleviates oxidative damage in poplar. Plant Physiol..

[77] Porth I., Klapšte J., Skyba O., Hannemann J., McKown A.D., Guy R.D., DiFazio S.P., Muchero W., Ranjan P., Tuskan G.A. (2013). Genome-wide association mapping for wood characteristics in P opulus identifies an array of candidate single nucleotide polymorphisms. New Phytol..

[78] Yoshimura K., Masuda A., Kuwano M., Yokota A., Akashi K. (2008). Programmed proteome response for drought avoidance/tolerance in the root of a C3 xerophyte (wild watermelon) under water deficits. Plant Cell Physiol..

[79] Liu W., Jiang Y., Jin Y., Wang C., Yang J., Qi H. (2021). Drought-induced ABA, H_2_O_2_ and JA positively regulate *CmCAD* genes and lignin synthesis in melon stems. BMC Plant Biol..

[80] Naseer S., Lee Y., Lapierre C., Franke R., Nawrath C., Geldner N. (2012). Casparian strip diffusion barrier in *Arabidopsis* is made of a lignin polymer without suberin. Proc. Natl. Acad. Sci. USA.

[81] Brunner I., Herzog C., Dawes M.A., Arend M., Sperisen C. (2015). How tree roots respond to drought. Front. Plant Sci..

[82] Lens F., Sperry J.S., Christman M.A., Choat B., Rabaey D., Jansen S. (2011). Testing hypotheses that link wood anatomy to cavitation resistance and hydraulic conductivity in the genus *Acer*. New Phytol..

[83] Choi S.J., Lee Z., Kim S., Jeong E., Shim J.S. (2023). Modulation of lignin biosynthesis for drought tolerance in plants. Front. Plant Sci..

[84] Hou N., Li C., He J., Liu Y., Yu S., Malnoy M., Mobeen Tahir M., Xu L., Ma F., Guan Q. (2022). MdMTA-mediated m^6^A modification enhances drought tolerance by promoting mRNA stability and translation efficiency of genes involved in lignin deposition and oxidative stress. New Phytol..

[85] Zhang X., Zou S., Yao C., Shan Y., Cai J., Li C., Wu J. (2025). ABA-induced PbrMYB8-PbrMYB169 module promotes lignin biosynthesis in corking disorder in pear fruit. Hortic. Plant J..

[86] Acosta-Motos J.R., Ortuño M.F., Bernal-Vicente A., Diaz-Vivancos P., Sanchez-Blanco M.J., Hernandez J.A. (2017). Plant responses to salt stress: Adaptive mechanisms. Agronomy.

[87] Yang Y., Guo Y. (2018). Elucidating the molecular mechanisms mediating plant salt-stress responses. New Phytol..

[88] Xie R., Pan X., Zhang J., Ma Y., He S., Zheng Y., Ma Y. (2018). Effect of salt-stress on gene expression in citrus roots revealed by RNA-seq. Funct. Integr. Genom..

[89] Krishnamurthy P., Ranathunge K., Franke R., Prakash H., Schreiber L., Mathew M. (2009). The role of root apoplastic transport barriers in salt tolerance of rice (*Oryza sativa* L.). Planta.

[90] Wu F., Qu D., Zhang X., Sun Y., Wang J., Zhu D., Yang L., Liu X., Tian W., Wang L. (2023). PaLectinL7 enhances salt tolerance of sweet cherry by regulating lignin deposition in connection with PaCAD1. Tree Physiol..

[91] Geng Y., Wu R., Wee C.W., Xie F., Wei X., Chan P.M.Y., Tham C., Duan L., Dinneny J.R. (2013). A spatio-temporal understanding of growth regulation during the salt stress response in *Arabidopsis*. Plant Cell.

[92] Yoshida T., Ohama N., Nakajima J., Kidokoro S., Mizoi J., Nakashima K., Maruyama K., Kim J.-M., Seki M., Todaka D. (2011). *Arabidopsis* HsfA1 transcription factors function as the main positive regulators in heat shock-responsive gene expression. Mol. Genet. Genom..

[93] Liu X., Chen H., Li S., Lecourieux D., Duan W., Fan P., Liang Z., Wang L. (2023). Natural variations of HSFA2 enhance thermotolerance in grapevine. Hortic. Res..

[94] Sharma A., Shahzad B., Rehman A., Bhardwaj R., Landi M., Zheng B. (2019). Response of phenylpropanoid pathway and the role of polyphenols in plants under abiotic stress. Molecules.

[95] Jing Y., Pei T., Li C., Wang D., Wang Q., Chen Y., Li P., Liu C., Ma F. (2023). Overexpression of the FERONIA receptor kinase MdMRLK2 enhances apple cold tolerance. Plant J..

[96] Finger-Teixeira A., Ferrarese M.d.L.L., Soares A.R., da Silva D., Ferrarese-Filho O. (2010). Cadmium-induced lignification restricts soybean root growth. Ecotoxicol. Environ. Saf..

[97] Vázquez S., Agha R., Granado A., Sarro M., Esteban E., Peñalosa J., Carpena R. (2006). Use of white lupin plant for phytostabilization of Cd and As polluted acid soil. Water Air Soil Pollut..

[98] Passardi F., Cosio C., Penel C., Dunand C. (2005). Peroxidases have more functions than a Swiss army knife. Plant Cell Rep..

[99] Elobeid M., Göbel C., Feussner I., Polle A. (2012). Cadmium interferes with auxin physiology and lignification in poplar. J. Exp. Bot..

[100] Dixon R.A., Achnine L., Kota P., Liu C.J., Reddy M.S., Wang L. (2002). The phenylpropanoid pathway and plant defence—A genomics perspective. Mol. Plant Pathol..

[101] Vanhaelewyn L., Van Der Straeten D., De Coninck B., Vandenbussche F. (2020). Ultraviolet radiation from a plant perspective: The plant-microorganism context. Front. Plant Sci..

[102] Henry-Kirk R.A., Plunkett B., Hall M., McGhie T., Allan A.C., Wargent J.J., Espley R.V. (2018). Solar UV light regulates flavonoid metabolism in apple (*Malus* × *domestica*). Plant Cell Environ..

[103] Duan S., Eom S.H. (2023). Regulation of anthocyanin and lignin contents in postharvest ‘Fuji’apple irradiated with UV-B. Sci. Hortic..

[104] Shi C.-H., Wang X.-Q., Zhang X.-Y., Shen L.-Y., Luo J., Zhang Y.-X. (2019). Response of fruit bagging to lignin biosynthesis and expression of related genes in fruit peel of sand pear (*Pyrus pyrifolia* Nakai) cv. Cuiguan. Hortscience.

[105] Mittler R., Vanderauwera S., Suzuki N., Miller G., Tognetti V.B., Vandepoele K., Gollery M., Shulaev V., Van Breusegem F. (2011). ROS signaling: The new wave?. Trends Plant Sci..

[106] Munzert K.S., Engelsdorf T. (2025). Plant cell wall structure and dynamics in plant–pathogen interactions and pathogen defence. J. Exp. Bot..

[107] Wilhelm E. (2024). Applications of Plant Tissue Culture for Studies of Fruit Tree Defense Mechanisms. Biological Control of Plant Diseases.

[108] Hou Y., Yu X., Chen W., Zhuang W., Wang S., Sun C., Cao L., Zhou T., Qu S. (2021). *MdWRKY75e* enhances resistance to Alternaria alternata in *Malus domestica*. Hortic. Res..

[109] Zhu L., Ni W., Liu S., Cai B., Xing H., Wang S. (2017). Transcriptomics analysis of apple leaves in response to Alternaria alternata apple pathotype infection. Front. Plant Sci..

[110] Rahman M.U., Liu X., Wang X., Fan B. (2024). Grapevine gray mold disease: Infection, defense and management. Hortic. Res..

[111] Li X., Li F., Wang P., Li H., Wang G., Wang S., Cao X., Sun J., Cao L., Zhang L. (2025). The resistance of litchi fruit to *Peronophythora litchii* is associated with lignin biosynthesis and reactive oxygen species metabolism. Sci. Hortic..

[112] Gallego-Giraldo L., Posé S., Pattathil S., Peralta A.G., Hahn M.G., Ayre B.G., Sunuwar J., Hernandez J., Patel M., Shah J. (2018). Elicitors and defense gene induction in plants with altered lignin compositions. New Phytol..

[113] Zhang L., Liu J., Cheng J., Sun Q., Zhang Y., Liu J., Li H., Zhang Z., Wang P., Cai C. (2022). *lncRNA7* and *lncRNA2* modulate cell wall defense genes to regulate cotton resistance to *Verticillium wilt*. Plant Physiol..

[114] Choi H.W., Kim Y.J., Lee S.C., Hong J.K., Hwang B.K. (2007). Hydrogen peroxide generation by the pepper extracellular peroxidase CaPO2 activates local and systemic cell death and defense response to bacterial pathogens. Plant Physiol..

[115] Boller T., Felix G. (2009). A renaissance of elicitors: Perception of microbe-associated molecular patterns and danger signals by pattern-recognition receptors. Annu. Rev. Plant Biol..

[116] Meng X., Zhang S. (2013). MAPK cascades in plant disease resistance signaling. Annu. Rev. Phytopathol..

[117] Li Y., Zhang D., Wang X., Bai F., Li R., Zhou R., Wu S., Fang Z., Liu W., Huang L. (2025). LACCASE35 enhances lignification and resistance against Pseudomonas syringae pv. actinidiae infection in kiwifruit. Plant Physiol..

[118] Torres M.A., Jones J.D., Dangl J.L. (2006). Reactive oxygen species signaling in response to pathogens. Plant Physiol..

[119] Bonasera J.M., Kim J.F., Beer S.V. (2006). PR genes of apple: Identification and expression in response to elicitors and inoculation with Erwinia amylovora. BMC Plant Biol..

[120] Xu P., Chen F., Mannas J.P., Feldman T., Sumner L.W., Roossinck M.J. (2008). Virus infection improves drought tolerance. New Phytol..

[121] Hipper C., Brault V., Ziegler-Graff V., Revers F. (2013). Viral and cellular factors involved in phloem transport of plant viruses. Front. Plant Sci..

[122] Lewis J.D., Knoblauch M., Turgeon R. (2022). The phloem as an arena for plant pathogens. Annu. Rev. Phytopathol..

[123] Hajimorad M., Eggenberger A., Hill J. (2005). Loss and gain of elicitor function of Soybean mosaic virus G7 provoking Rsv1-mediated lethal systemic hypersensitive response maps to P3. J. Virol..

[124] Allen H., Zeef L., Morreel K., Goeminne G., Kumar M., Gomez L.D., Dean A.P., Eckmann A., Casiraghi C., McQueen-Mason S.J. (2022). Flexible and digestible wood caused by viral-induced alteration of cell wall composition. Curr. Biol..

[125] Gao D., Xing F., Yan Q., Zhang Z., Zhan B., Lu M., Ma Y., Wang H., Li S., Xie J. (2025). Transcriptome Analysis of Apple Leaves with Apple Necrotic Mosaic Virus-Associated Mosaic Symptoms. Plants.

[126] Dhakshinamoorthy S., Mariama K., Elsen A., De Waele D. (2014). Phenols and lignin are involved in the defence response of banana (*Musa*) plants to Radopholus similis infection. Nematology.

[127] War A.R., Paulraj M.G., Ahmad T., Buhroo A.A., Hussain B., Ignacimuthu S., Sharma H.C. (2012). Mechanisms of plant defense against insect herbivores. Plant Signal. Behav..

[128] Dar T.A., Uddin M., Khan M.M.A., Hakeem K., Jaleel H. (2015). Jasmonates counter plant stress: A review. Environ. Exp. Bot..

[129] Wu J., Baldwin I.T. (2010). New insights into plant responses to the attack from insect herbivores. Annu. Rev. Genet..

[130] Jardim B.C., Perdizio V.A., Berbert-Molina M.A., Rodrigues D.C., Botelho-Junior S., Vicente A.C., Hansen E., Otsuki K., Urmenyi T.P., Jacinto T. (2010). Herbivore response in passion fruit (*Passiflora edulis* Sims) plants: Induction of lipoxygenase activity in leaf tissue in response to generalist and specialist insect attack. Protein Pept. Lett..

[131] Heil M., Karban R. (2010). Explaining evolution of plant communication by airborne signals. Trends Ecol. Evol..

[132] Wang W., Zhou X., Hu Q., Wang Q., Zhou Y., Yu J., Ge S., Zhang L., Guo H., Tang M. (2025). Lignin Metabolism Is Crucial in the Plant Responses to Tambocerus elongatus (Shen) in *Camellia sinensis* L. Plants.

[133] Smant G., Jones J. (2011). Suppression of plant defences by nematodes. Genomics and Molecular Genetics of Plant-Nematode Interactions.

[134] Nishad R., Ahmed T., Rahman V.J., Kareem A. (2020). Modulation of plant defense system in response to microbial interactions. Front. Microbiol..

[135] Huot B., Yao J., Montgomery B.L., He S.Y. (2014). Growth–defense tradeoffs in plants: A balancing act to optimize fitness. Mol. Plant.

[136] Su X., Zhao Y., Wang H., Li G., Cheng X., Jin Q., Cai Y. (2019). Transcriptomic analysis of early fruit development in Chinese white pear (*Pyrus bretschneideri* Rehd.) and functional identification of PbCCR1 in lignin biosynthesis. BMC Plant Biol..

[137] Ma N., Xiao Z., Lu L., Zhang H., Liu C., Xu Y., Qi Y., Gao Z. (2025). Multi-Omics Dissection of Gene–Metabolite Networks Underlying Lenticel Spot Formation via Cell-Wall Deposition in Pear Peel. Agronomy.

[138] Li X., Huang H., Rizwan H.M., Wang N., Jiang J., She W., Zheng G., Pan H., Guo Z., Pan D. (2022). Transcriptome analysis reveals candidate lignin-related genes and transcription factors during fruit development in pomelo (*Citrus maxima*). Genes.

[139] Ton J., Flors V., Mauch-Mani B. (2009). The multifaceted role of ABA in disease resistance. Trends Plant Sci..

[140] Zhong R., Ye Z.-H. (2009). Transcriptional regulation of lignin biosynthesis. Plant Signal. Behav..

[141] Sulis D.B., Wang J.P. (2020). Regulation of lignin biosynthesis by post-translational protein modifications. Front. Plant Sci..

[142] Yang J., Zhang Y., Jia J., Wang C., Fu Y. (2025). Flavonoid-Lignin Crosstalk: Engineering Metabolic Flux for Optimised Plant Growth and Stress Resilience. Plant Cell Environ..

[143] Ali M., Kaderbek T., Khan M.A., Skalicky M., Brestic M., Elsabagh M., El Sabagh A. (2025). Biosynthesis and multifaceted roles of reactive species in plant defense mechanisms during environmental cues. Plant Stress.

[144] Mittler R. (2017). ROS are good. Trends Plant Sci..

[145] Yadav V., Wang Z., Wei C., Amo A., Ahmed B., Yang X., Zhang X. (2020). Phenylpropanoid pathway engineering: An emerging approach towards plant defense. Pathogens.

[146] Zhao Q., Dixon R.A. (2014). Altering the cell wall and its impact on plant disease: From forage to bioenergy. Annu. Rev. Phytopathol..

[147] Wei Y., Liu Z., Lv T., Xu Y., Wei Y., Liu W., Liu L., Wang A., Li T. (2023). Ethylene enhances MdMAPK3-mediated phosphorylation of MdNAC72 to promote apple fruit softening. Plant Cell.

[148] Zhao L., Cassan-Wang H., Zhao Y., Bao Y., Hou Y., Liu Y., Wu Z., Bouzayen M., Zheng Y., Jin P. (2025). Calcium-dependent protein kinase PpCDPK29-mediated Ca^2+^-ROS signal and PpHSFA2a phosphorylation regulate postharvest chilling tolerance of peach fruit. Plant Biotechnol. J..

[149] Lu S., Li Q., Wei H., Chang M.-J., Tunlaya-Anukit S., Kim H., Liu J., Song J., Sun Y.-H., Yuan L. (2013). Ptr-miR397a is a negative regulator of laccase genes affecting lignin content in *Populus trichocarpa*. Proc. Natl. Acad. Sci. USA.

[150] Wang C.Y., Zhang S., Yu Y., Luo Y.C., Liu Q., Ju C., Zhang Y.C., Qu L.H., Lucas W.J., Wang X. (2014). MiR397b regulates both lignin content and seed number in *Arabidopsis* via modulating a laccase involved in lignin biosynthesis. Plant Biotechnol. J..

[151] Jeena G.S., Singh N., Shikha, Shukla R.K. (2022). An insight into microRNA biogenesis and its regulatory role in plant secondary metabolism. Plant Cell Rep..

[152] Sharma A., Badola P.K., Gautam H., Trivedi P.K. (2023). Heterologous expression of *Arabidopsis* miR858 modulates biosynthesis of secondary metabolites and affects drought tolerance in tobacco. Plant Cell Tissue Organ Cult. (PCTOC).

[153] Barciszewska-Pacak M., Milanowska K., Knop K., Bielewicz D., Nuc P., Plewka P., Pacak A.M., Vazquez F., Karlowski W., Jarmolowski A. (2015). *Arabidopsis* microRNA expression regulation in a wide range of abiotic stress responses. Front. Plant Sci..

[154] Sanz-Carbonell A., Marques M.C., Bustamante A., Fares M.A., Rodrigo G., Gomez G. (2019). Inferring the regulatory network of the miRNA-mediated response to biotic and abiotic stress in melon. BMC Plant Biol..

[155] Law J.A., Jacobsen S.E. (2010). Establishing, maintaining and modifying DNA methylation patterns in plants and animals. Nat. Rev. Genet..

[156] Chinnusamy V., Zhu J.-K. (2009). Epigenetic regulation of stress responses in plants. Curr. Opin. Plant Biol..

[157] Latzel V., Allan E., Bortolini Silveira A., Colot V., Fischer M., Bossdorf O. (2013). Epigenetic diversity increases the productivity and stability of plant populations. Nat. Commun..

[158] Helfer A., Nusinow D.A., Chow B.Y., Gehrke A.R., Bulyk M.L., Kay S.A. (2011). LUX ARRHYTHMO encodes a nighttime repressor of circadian gene expression in the *Arabidopsis* core clock. Curr. Biol..

[159] Rogers L.A., Dubos C., Cullis I.F., Surman C., Poole M., Willment J., Mansfield S.D., Campbell M.M. (2005). Light, the circadian clock, and sugar perception in the control of lignin biosynthesis. J. Exp. Bot..

[160] Xie Q., Wang P., Liu X., Yuan L., Wang L., Zhang C., Li Y., Xing H., Zhi L., Yue Z. (2014). LNK1 and LNK2 are transcriptional coactivators in the *Arabidopsis* circadian oscillator. Plant Cell.

[161] Liu J., Huang Q., Kang P., Liang L., Chen J. (2019). Lignin accumulation in three pumelo cultivars in association with sucrose and energy depletion. Biomolecules.

[162] Wang Q., Hu J., Yang T., Chang S. (2021). Anatomy and lignin deposition of stone cell in Camellia oleifera shell during the young stage. Protoplasma.

[163] Xue C., Yao J.-L., Xue Y.-S., Su G.-Q., Wang L., Lin L.-K., Allan A.C., Zhang S.-L., Wu J. (2019). PbrMYB169 positively regulates lignification of stone cells in pear fruit. J. Exp. Bot..

[164] Herremans E., Verboven P., Hertog M.L., Cantre D., Van Dael M., De Schryver T., Van Hoorebeke L., Nicolaï B.M. (2015). Spatial development of transport structures in apple (*Malus* × *domestica* Borkh.) fruit. Front. Plant Sci..

[165] Torres C.A., Azocar C., Ramos P., Pérez-Díaz R., Sepulveda G., Moya-León M.A. (2020). Photooxidative stress activates a complex multigenic response integrating the phenylpropanoid pathway and ethylene, leading to lignin accumulation in apple (*Malus domestica* Borkh.) fruit. Hortic. Res..

[166] Tacken E., Ireland H., Gunaseelan K., Karunairetnam S., Wang D., Schultz K., Bowen J., Atkinson R.G., Johnston J.W., Putterill J. (2010). The role of ethylene and cold temperature in the regulation of the apple *POLYGALACTURONASE1* gene and fruit softening. Plant Physiol..

[167] Janssen B.J., Thodey K., Schaffer R.J., Alba R., Balakrishnan L., Bishop R., Bowen J.H., Crowhurst R.N., Gleave A.P., Ledger S. (2008). Global gene expression analysis of apple fruit development from the floral bud to ripe fruit. BMC Plant Biol..

[168] Downey M.O., Harvey J.S., Robinson S.P. (2003). Analysis of tannins in seeds and skins of Shiraz grapes throughout berry development. Aust. J. Grape Wine Res..

[169] Pilati S., Bagagli G., Sonego P., Moretto M., Brazzale D., Castorina G., Simoni L., Tonelli C., Guella G., Engelen K. (2017). Abscisic acid is a major regulator of grape berry ripening onset: New insights into ABA signaling network. Front. Plant Sci..

[170] Ma Y., Luo J., Xu Y. (2019). Co-preparation of pectin and cellulose from apple pomace by a sequential process. J. Food Sci. Technol..

[171] Li F., Tahir M.M., Yang C., Weng Z., Zhu W., Zhang Y., Zhou K., Deng Q., Qian M., Wu H. (2025). Postharvest UV-A treatment promotes mango fruit pigmentation and ripening in a dose-dependent manner. Postharvest Biol. Technol..

[172] Cai C., Chen K., Xu W., Zhang W., Li X., Ferguson I. (2006). Effect of 1-MCP on postharvest quality of loquat fruit. Postharvest Biol. Technol..

[173] Zeng J.K., Li X., Xu Q., Chen J.Y., Yin X.R., Ferguson I.B., Chen K.S. (2015). Ej AP 2-1, an AP 2/ERF gene, is a novel regulator of fruit lignification induced by chilling injury, via interaction with Ej MYB transcription factors. Plant Biotechnol. J..

[174] Zeng J.K., Li X., Zhang J., Ge H., Yin X.R., Chen K.s. (2016). Regulation of loquat fruit low temperature response and lignification involves interaction of heat shock factors and genes associated with lignin biosynthesis. Plant Cell Environ..

[175] Xu R., Zhou J., Deng L., Zhang S., Golding J.B., Wang B. (2025). Transcriptomics integrated with metabolomics analysis of cold-induced lenticel disorder via the lignin pathway upon postharvest ‘Xinli No. 7’pear fruit. Postharvest Biol. Technol..

[176] Zhang J., Yin X.-R., Li H., Xu M., Zhang M.-X., Li S.-J., Liu X.-F., Shi Y.-N., Grierson D., Chen K.-S. (2020). ETHYLENE RESPONSE FACTOR39–MYB8 complex regulates low-temperature-induced lignification of loquat fruit. J. Exp. Bot..

[177] Bhardwaj R., Aghdam M.S., Arnao M.B., Brecht J.K., Fawole O.A., Pareek S. (2022). Melatonin alleviates chilling injury symptom development in mango fruit by maintaining intracellular energy and cell wall and membrane stability. Front. Nutr..

[178] Watkins C.B. (2006). The use of 1-methylcyclopropene (1-MCP) on fruits and vegetables. Biotechnol. Adv..

[179] Ming M., Long H., Ye Z., Pan C., Chen J., Tian R., Sun C., Xue Y., Zhang Y., Li J. (2022). Highly efficient CRISPR systems for loss-of-function and gain-of-function research in pear calli. Hortic. Res..

[180] Wang H., Sun L., Zhang S., Li D. Copper Regulates the Expressions of Mdlac7 and Mdccr1 to Promote the Biosynthesis of Lignin in Apple Peel. https://papers.ssrn.com/sol3/papers.cfm?abstract_id=4340701.

[181] Chen E., Jia J., Sun J., Chen X., Li F., Li X. (2025). Revealing the regulation of HuMYBS3 on HuCOMT1 and HuCHI in response to biotic and abiotic stress through trypsin-induced resistance in pitaya using bulk and single-cell transcriptome profiles. Plant Growth Regul..

[182] Liu H., Zhou H., Ye H., Li M., Ma J., Xi R., He X., Zhao P. (2025). Integrated multi-omics analyses provide new insights into genomic variation landscape and regulatory network candidate genes associated with walnut endocarp. Plant J..

[183] Hu S., Liu Y., Zhao Y., Li B., Shen Y., Mimata Y., Zhang K., Hao P., Shi J., Luo Z. (2026). Comparative evaluation of reference genomes and cell-type annotation frameworks for single-nucleus transcriptomic analysis in apple. Agric. Commun..

