# Supplementary material for: The Dual Role of Lignin in Fruit Trees: Unraveling Regulatory Networks from Stress Resilience to Quality Control
Source: Plants (Basel). 2026 Jul 22;15(14):2244. doi: 10.3390/plants15142244 (PMC13431395; doi:10.3390/plants15142244)
Supplement: Supplementary file 1 [file plants-15-02244-s001.zip › Supplementary data/Table S1.pdf]

Table S1: List of abbreviations

Abbreviation	Full Form
4CL	4-Coumarate:CoA Ligase
ABA	Abscisic Acid
ABC	ATP-Binding Cassette
ANS	Anthocyanidin Synthase
ApNMV	Apple Necrotic Mosaic Virus
ARWV	Apple Rubbery Wood Virus
ATP	Adenosine Triphosphate
BES1	BRI1-EMS-SUPPRESSOR 1
BR	Brassinosteroid
BRI1	Brassinosteroid Insensitive 1
BZR1	BRASSINAZOLE-RESISTANT 1
C3H	p-Coumarate 3-Hydroxylase
C4H	Cinnamate 4-Hydroxylase
CAD	Cinnamyl Alcohol Dehydrogenase
CAT	Catalase
CBF	C-Repeat Binding Factor

Abbreviation	Full Form
CCA1	CIRCADIAN CLOCK ASSOCIATED 1
CCoAOMT	Caffeoyl-CoA O-Methyltransferase
CCR	Cinnamoyl-CoA Reductase
CDPK	Calcium-Dependent Protein Kinase
COI1	Coronatine Insensitive 1
COMT	Caffeic Acid O-Methyltransferase
CRISPR/Cas9	Clustered Regularly Interspaced Short Palindromic Repeats/CRISPR-Associated Protein 9
DAFB	Days After Full Bloom
DFR	Dihydroflavonol 4-Reductase
DNA	Deoxyribonucleic Acid
DREB	Dehydration-Responsive Element-Binding Protein
ER	Endoplasmic Reticulum
ERF	Ethylene Response Factor
F5H	Ferulate 5-Hydroxylase
FLS	Flavonol Synthase
G	Guaiacyl (monolignol unit)
GA	Gibberellic Acid

Abbreviation	Full Form
GWAS	Genome-Wide Association Study
H	p-Hydroxyphenyl (monolignol unit)
H_2O_2	Hydrogen Peroxide
HCT	p-Coumaroyl Shikimate/Quinate Hydroxycinnamoyltransferase
HDA	Histone Deacetylase
HSF	Heat Shock Factor
HY5	ELONGATED HYPOCOTYL 5
JA	Jasmonic Acid
JAZ	JASMONATE ZIM-DOMAIN
LAC	Laccase
LHY	LATE ELONGATED HYPOCOTYL
lncRNA	Long Non-Coding RNA
LOX	Lipoxygenase
m^6A	N6-Methyladenosine
MAPK	Mitogen-Activated Protein Kinase
miRNA	MicroRNA
MYB	Myeloblastosis (transcription factor family)

Abbreviation	Full Form
NAC	NAM, ATAF1/2, CUC2 (transcription factor family)
NADPH	Nicotinamide Adenine Dinucleotide Phosphate
PAL	Phenylalanine Ammonia-Lyase
PAMP	Pathogen-Associated Molecular Pattern
POD	Peroxidase
PP2C	Protein Phosphatase 2C
PR	Pathogenesis-Related
PRR	Pattern Recognition Receptor
PRX	Peroxidase
Psa	Pseudomonas syringae pv. actinidiae
PYR/PYL/RCAR	Pyrabactin Resistance/PYR-Like/Regulatory Component of ABA Receptor
RBOH	Respiratory Burst Oxidase Homolog
RISC	RNA-Induced Silencing Complex
RNS	Reactive Nitrogen Species
RNA	Ribonucleic Acid
ROS	Reactive Oxygen Species
S	Syringyl (monolignol unit)

Abbreviation	Full Form
SA	Salicylic Acid
SAR	Systemic Acquired Resistance
SnRK2	SNF1-Related Protein Kinase 2
SOD	Superoxide Dismutase
TF	Transcription Factor
TIR1/AFB	Transport Inhibitor Response 1/Auxin Signaling F-Box
UFGT	UDP-Glucose:Flavonoid 3-O-Glucosyltransferase
UPS	Ubiquitin-Proteasome System
UV	Ultraviolet
UVR8	UV Resistance Locus 8
vasiRNA	Virus-Activated Small Interfering RNA
VOC	Volatile Organic Compound
WGCNA	Weighted Gene Co-Expression Network Analysis
WRKY	WRKY (transcription factor family named after conserved WRKYGQK motif)
