# Supplementary material for: The Dual Role of Lignin in Fruit Trees: Unraveling Regulatory Networks from Stress Resilience to Quality Control
Source: Plants (Basel). 2026 Jul 22;15(14):2244. doi: 10.3390/plants15142244 (PMC13431395; doi:10.3390/plants15142244)
Supplement: Supplementary file 1 [file plants-15-02244-s001.zip › Supplementary data/Table S2.pdf]

Table S2: Catalog of reported lignin-related genes in fruit tree species

Gene name	Species	Function/regulatory role	Key references
**Biosynthetic enzymes**			
MdPAL	Apple (*Malus* × *domestica*)	Phenylalanine ammonia-lyase; drought/salt-induced lignin deposition	[16]
MdC4H	Apple (*Malus* × *domestica*)	Cinnamate 4-hydroxylase; cold acclimation and drought response	[16]
Md4CL	Apple (*Malus* × *domestica*)	4-Coumarate:CoA ligase; aluminum stress-induced lignification	[16]
MdCCR	Apple (*Malus* × *domestica*)	Cinnamoyl-CoA reductase; heavy metal stress response	[16]
MdCAD	Apple (*Malus* × *domestica*)	Cinnamyl alcohol dehydrogenase; drought tolerance	[43]
MdCCR1	Apple (*Malus* × *domestica*)	Cinnamoyl-CoA reductase; validated by CRISPR/Cas9 knockout	[179]
MdLAC7	Apple (*Malus* × *domestica*)	Laccase; defense lignification against *Alternaria alternata*	[108]
CsPAL	Citrus (*Citrus spp.*)	Phenylalanine ammonia-lyase; salt stress-induced expression	[88]
CsC4H	Citrus (*Citrus spp.*)	Cinnamate 4-hydroxylase; salinity tolerance	[88]

Gene name	Species	Function/regulatory role	Key references
Cs4CL	Citrus (*Citrus spp.*)	4-Coumarate:CoA ligase; salt stress response	[88]
CgCCoAOMT	Pomelo (*Citrus grandis*)	Caffeoyl-CoA O-methyltransferase; juice sac lignification	[46]
CgC3H	Pomelo (*Citrus grandis*)	p-Coumarate 3-hydroxylase; juice sac lignification	[46]
PbPAL1	Pear (*Pyrus bretschneideri*)	Phenylalanine ammonia-lyase; stone cell lignification	[163]
Pb4CL1	Pear (*Pyrus bretschneideri*)	4-Coumarate:CoA ligase; stone cell development	[163]
PbCAD1	Pear (*Pyrus bretschneideri*)	Cinnamyl alcohol dehydrogenase; stone cell lignification	[163]
Pb4CL4	Pear (*Pyrus bretschneideri*)	4-Coumarate:CoA ligase; stone cell regulation via PbrNSC	[47]
PbLAC4	Pear (*Pyrus bretschneideri*)	Laccase; stone cell lignification	[47]
PbCCR1	Pear (*Pyrus bretschneideri*)	Cinnamoyl-CoA reductase; key lignin gene during early fruit development	[136]
EjPAL1	Loquat (*Eriobotrya japonica*)	Phenylalanine ammonia-lyase; chilling-induced lignification	[174]

Gene name	Species	Function/regulatory role	Key references
Ej4CL1	Loquat (*Eriobotrya japonica*)	4-Coumarate:CoA ligase; flesh lignification at 0°C	[13]
EjCAD1	Loquat (*Eriobotrya japonica*)	Cinnamyl alcohol dehydrogenase; woolly texture development	[13]
CmCAD1, CmCAD 2, CmCAD 3, CmCAD 5	Melon (*Cucumis melo*)	Cinnamyl alcohol dehydrogenase isoforms; drought-induced stem lignification	[80]
CmPAL	Melon (*Cucumis melo*)	Phenylalanine ammonia-lyase; drought stress response	[80]
CmPOD	Melon (*Cucumis melo*)	Peroxidase; drought-induced lignification	[80]
CmLAC	Melon (*Cucumis melo*)	Laccase; drought-induced lignification	[80]
AcLac35	Kiwifruit (*Actinidia chinensis*)	Laccase; resistance to *Pseudomonas syringae* pv. *actinidiae*	[117]
PaCAD1	Sweet cherry (*Prunus avium*)	Cinnamyl alcohol dehydrogenase; phosphorylation by PaLectinL7 promotes lignification	[90]
HuCOMT1	Pitaya (*Hylocereus undatus*)	Caffeic acid O-methyltransferase; lignin biosynthesis in exocarp	[180]

Gene name	Species	Function/regulatory role	Key references
VvC4H	Grapevine (*Vitis vinifera*)	Cinnamate 4-hydroxylase; stress-responsive lignification via VvWRKY2	[52]
**Transporters & glucosidases**			
PgABCG9	Pomegranate (*Punica granatum*)	ABC transporter; negative regulator of lignin accumulation	[30]
MdABCG25	Apple (*Malus* × *domestica*)	ABC transporter; stress-inducible monolignol transport	[31]
PbBGLU16	Pear (*Pyrus bretschneideri*)	β-Glucosidase; hydrolyzes monolignol glucosides; promotes stone cell lignification	[35]
**Transcription factors - nac family**			
MdSND1	Apple (*Malus* × *domestica*)	NAC TF; initiates lignin biosynthesis; enhances salt/osmotic stress tolerance	[43]
CgNAC043	Pomelo (*Citrus grandis*)	NAC TF; activates *CgMYB46* and lignin genes; juice sac lignification	[46]
PbrNSC	Pear (*Pyrus bretschneideri*)	NAC TF; promotes stone cell lignification via *PbrMYB169*, *Pbr4CL4*, *PbrLAC4*	[47]
EjNAC5	Loquat (*Eriobotrya japonica*)	NAC TF; regulates chilling-induced lignification; promoter methylation regulates expression	[76]

Gene name	Species	Function/regulatory role	Key references
MdNAC72	Apple (*Malus* × *domestica*)	NAC TF; phosphorylation by *MdMAPK3* promotes fruit softening	[147]
**Transcription factors - myb family (activators)**			
MdMYB46	Apple (*Malus* × *domestica*)	R2R3-MYB; activates lignin genes; salt/osmotic stress tolerance	[45]
MdMYB83	Apple (*Malus* × *domestica*)	R2R3-MYB; secondary wall formation; stress-induced lignification	[43]
MdMYB88	Apple (*Malus* × *domestica*)	R2R3-MYB; phenylpropanoid metabolism; pathogen and drought resistance; UPS-regulated	[73]
MdMYB124	Apple (*Malus* × *domestica*)	R2R3-MYB; phenylpropanoid metabolism; pathogen and drought resistance	[73]
CgMYB46	Pomelo (*Citrus grandis*)	R2R3-MYB; activated by CgNAC043; lignin biosynthesis	[46]
PbrMYB169	Pear (*Pyrus bretschneideri*)	R2R3-MYB; positive regulator of stone cell lignification	[163]
PbrMYB8	Pear (*Pyrus bretschneideri*)	R2R3-MYB; ABA-induced module with PbrMYB169; corking disorder	[85]
PbrMYB14	Pear (*Pyrus bretschneideri*)	R2R3-MYB; integrates lignin and SA biosynthesis; resistance to *Alternaria alternata*	[63]

Gene name	Species	Function/regulatory role	Key references
PgMYB2	*Panax ginseng*	R2R3-MYB; MeJA-responsive; secondary metabolite regulation (conserved module in fruit trees)	[62]
HuMYBS3	Pitaya (*Hylocereus undatus*)	MYB TF; differential activation of lignin (*HuCOMT1*) and flavonoid (*HuCHI*) pathways	[180]
MdMYBPA1	Apple (*Malus* × *domestica*)	MYB TF; interacts with MdMRLK2; cold tolerance and anthocyanin regulation	[95]
**Transcription factors - wrky family**			
PbWRKY24	Pear (*Pyrus bretschneideri*)	WRKY TF; promotes russet skin lignification; UV-B-induced	[50]
PlWRKY29	Herbaceous peony (*Paeonia lactiflora*)	WRKY TF; JA/SA signaling integration; lignin regulation under stress	[51]
VvWRKY2	Grapevine (*Vitis vinifera*)	WRKY TF; binds *VvC4H* promoter; stress-responsive lignification	[52]
MdWRKY75e	Apple (*Malus* × *domestica*)	WRKY TF; JA-responsive; activates *MdLAC7* for defense lignification	[108]
MdWRKY9	Apple (*Malus* × *domestica*)	WRKY TF; phosphorylated by MdMAPK6; regulates fruit ripening	[147]

Gene name	Species	Function/regulatory role	Key references
**Transcription factors - erf/ap2 family**			
MdERF114	Apple (*Malus × domestica*)	ERF TF; binds *MdPRX63* promoter; enhances resistance to *Fusarium solani*	[53]
EjAP2-1	Loquat (*Eriobotrya japonica*)	AP2/ERF TF; represses lignification via interaction with EjMYB TFs	[173]
EjHSF3	Loquat (*Eriobotrya japonica*)	Heat shock factor; activates lignin biosynthesis under cold stress	[174]
VvHSFA2	Grapevine (*Vitis vinifera*)	Heat shock factor; thermotolerance and lignin regulation	[93]
**Post-transcriptional & epigenetic regulators**			
miR397	Pear, other fruit trees	miRNA; targets *LAC* genes; modulates lignin content	[66]
MdMTA	Apple (*Malus × domestica*)	m^6A RNA methyltransferase; stabilizes lignin and ROS-scavenging transcripts under drought	[84]
**Receptor kinases & signaling**			

Gene name	Species	Function/regulatory role	Key references
PaLectinL7	Sweet cherry (*Prunus avium*)	Lectin receptor-like kinase; salt tolerance; promotes lignin deposition; phosphorylates PaCAD1	[90]
MdMRLK2	Apple (*Malus* × *domestica*)	FERONIA-family receptor-like kinase; cold-induced; promotes lignin/cell wall accumulation	[95]
MdMAPK3	Apple (*Malus* × *domestica*)	Mitogen-activated protein kinase; phosphorylates MdNAC72; fruit softening	[147]
MdMAPK6	Apple (*Malus* × *domestica*)	Mitogen-activated protein kinase; phosphorylates MdWRKY9; ripening regulation	[147]
PpCDPK29	Peach (*Prunus persica*)	Calcium-dependent protein kinase; Ca^{2+}-ROS signaling; phosphorylates PpHSFA2a; chilling tolerance	[148]
